# Asymmetric Total Synthesis of Four Stereoisomers of the Sex Pheromone of the Western Corn Rootworm

**DOI:** 10.3390/molecules23030667

**Published:** 2018-03-15

**Authors:** Zhi-Feng Sun, Tao Zhang, Jinyang Liu, Zhen-Ting Du, Huaiji Zheng

**Affiliations:** 1 Shaanxi Key Laboratory of Natural Products and Chemical Biology, College of Chemistry and Pharmacy, North West Agriculture and Forestry University, Yangling 712100, China; sunzhifeng2018@163.com (Z.-F.S.); fuzitong@163.com (T.Z.); m17792029896@163.com (J.L.); 2 Shaanxi Key Laboratory for Catalysis, College of Chemical and Environment Science, Shaanxi University of Technology, Hanzhong 723001, China; 3 Key Laboratory of Botanical Pesticide R&D in Shaanxi Province, Yangling 712100, China

**Keywords:** pheromone, west corn rootworm, Evans methylation, Julia-Kocienski olefination, convergent synthesis

## Abstract

A convergent synthesis of four stereoisomers of the sex pheromone of the western corn rootworm (8-methyldecan-2-yl propionate, **1**) from commercially available chiral starting materials is reported. The key step was Julia–Kocienski olefination between chiral BT-sulfone and chiral aldehyde. This synthetic route provided the four stereoisomers of **1** in 24–29% total yield via a six-step sequence. The simple scale-up strategy provides a new way to achieve the asymmetric synthesis of the sex pheromone.

## 1. Introduction

**Figure 1:**
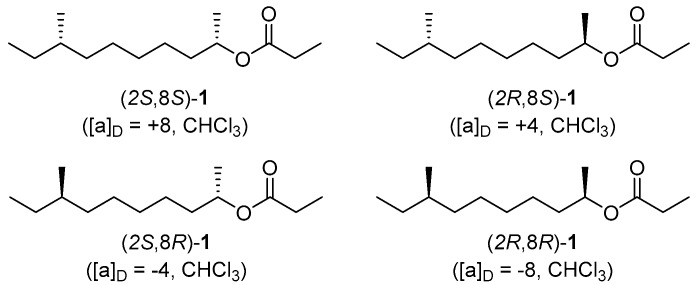
Four stereoisomers of the sex pheromone of the western corn rootworm (**1**).

Modern agriculture means finding ways to increase production while being friendly to the environment, i.e., sustainable development. Sex pheromone traps, as a simple, natural, and species-specific method have been adopted in agriculture to reduce the population densities of pest and decrease pesticide use [1,2,3]. The female-produced sex pheromone of the western corn rootworm (*Diabrotica virgifera virgifera* Le Conte) was isolated and identified as the structure of 8-methyldecan-2-yl propionate (**1**) (Figure 1) [4,5]. Different species of corn rootworm in America have different responses to the four possible stereoisomers of chiral **1 [6,7,8]**. As the western corn rootworm is spreading around the world, we urgently need to know how the male corn rootworm from other regions, especially from Europe and Asia, respond to these isomers. In order to obtain an abundant supply of enantiopure compounds for in-depth studies, we developed a convergent synthetic route to the four stereoisomers.

A number of different synthetic routes to **1** have been reported [5,9,10,11,12]. The enantioselective syntheses of the four isomers was exploited by the chiral auxiliary strategy and HPLC resolution of diastereomers [13], enzyme-catalyzed reduction [14], and Sharpless asymmetric dihydroxylation [15]. Others focused on the synthesis of one or two enantiomers. In 1984, Mori synthesized (*2S*,8*R*)-**1** and (*2R*,8*R*)-**1** via an alkylation of 1,3-dithiane strategy [16]. Ferreira synthesized (*2S*,8*S*)-**1** and (*2S*,8*R*)-**1** by a remote-controlled diastereoselective conjugated addition [17]. (*2R*,8*R*)-**1** was also obtained over an eight-step sequence from a proper diepoxide [18].

Recently, we have established a common route to generate the methyl-branched chiral center by Evans methylation and extend the carbon chain by the Julia–Kocienski coupling reaction in the pheromone synthesis [19]. Herein, a convergent strategy for the synthesis of four stereoisomers of **1** from commercially available chiral starting materials is reported, using Julia–Kocienski olefination [20] as a key step.

## 2. Results and Discussion

**Figure 2:**
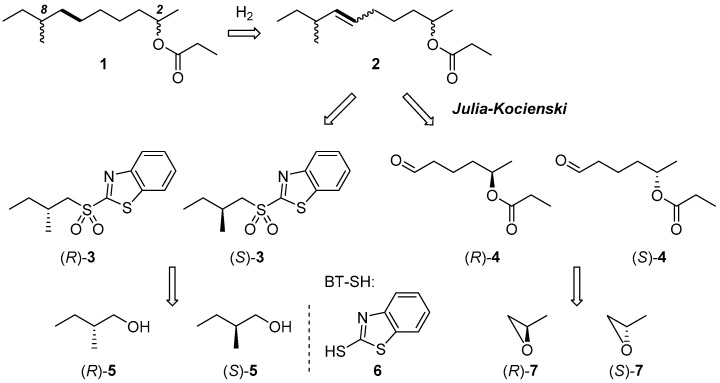
Retrosynthetic analysis of **1** based on Julia–Kocienski olefination.

A retrosynthetic analysis of the target molecule **1** is outlined in Figure 2. Intermediate **2** could be transformed into compound **1** via a catalytic hydrogenation process. Previously, a Julia–Kocienski coupling between chiral BT-sulfone **3** and chiral aldehyde **4** could provide intermediate **2**. Chiral **3** could be obtained from (*R*) or (*S*)-2-methyl-1-butanol (**5**), while chiral **4** could be prepared from (*R*) or (*S*)-2-methyloxirane (**7**).

**Scheme 1:**
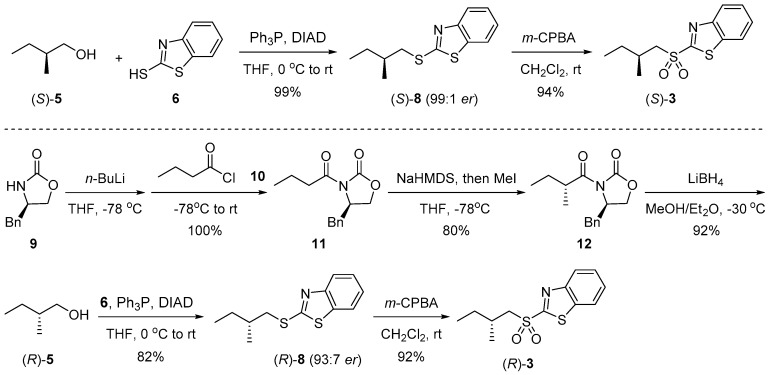
Synthesis of chiral sulfones (*S*)-**3** and (*R*)-**3**.

The BT-sulfone (*S*)-**3** was synthesized in high yield from commercially available chiral alcohol (*S*)-**5** via Mitsunobu reaction [21] and further *m*-CPBA oxidation (Scheme 1). Meanwhile, (*R*)-**3** was synthesized from (*R*)-**5** with the same reactions. However, the expensive starting material (*R*)-**5** was synthesized in 3 steps from Evans auxiliary [22] **9** with *R* configuration [23,24,25,26]. The enantioselectivity of Evans methylation was measured to be 93:7 *er* value for the derivative (*R*)-**8** by HPLC on a chiral OD-H column (See the Supplementary Materials).

**Scheme 2:**
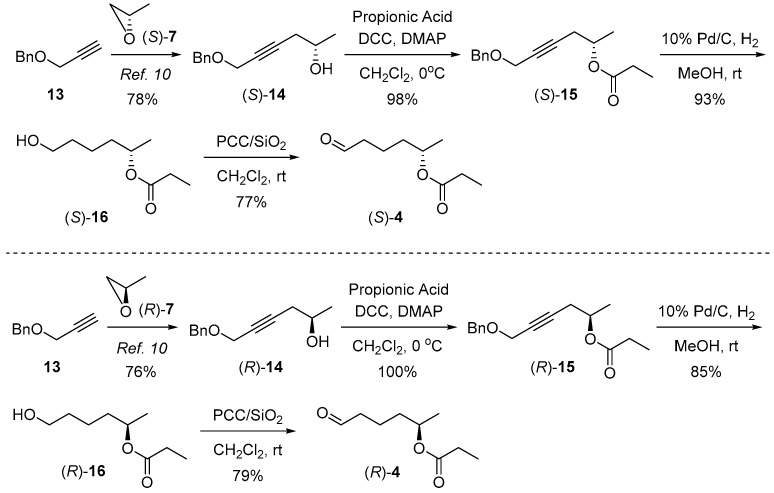
Synthesis of chiral aldehydes (*S*)-**4** and (*R*)-**4**.

The synthesis of chiral aldehyde (*S*)-**4** was started from (*S*)-2-methyloxirane ((*S*)-**7**), as shown in Scheme 2. Alcohol (*S*)-**14** was obtained according to the literature [27,28,29,30], and then esterified with propionic acid using DCC (*N*,*N*′-dicyclohexylcarbodiimide) as dehydrating agent. Catalytic hydrogenation of the resulting ester (*S*)-**15** directly provided hydroxyester (*S*)-**16**, as a result of the simultaneous removal of the benzyl protecting group and the hydrogenation of the carbon–carbon triple bond. Finally, oxidation of (*S*)-**16** by PCC (pyridinium chlorochromate), [31] provided (*S*)-**4** in a 55% total yield. Compound (*R*)-**4** was similarly obtained from (*R*)-**7** with a 46% global yield, via compounds (*R*)-**14**, (*R*)-**15**, and (*R*)-**16**.

**Scheme 3:**
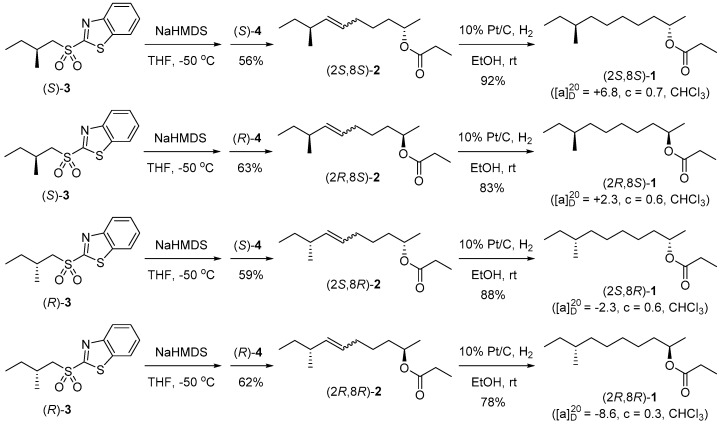
Total synthesis of the four stereoisomers of **1**.

Finally, compounds **1** were obtained by a Julia–Kocienski coupling of proper precursor **3** and **4**, followed by catalytic hydrogenation of the resulting compound **2**, under the conditions as stated in Scheme 3. The hydrogenation was catalyzed by Pt/C to avoid racemization of the allylic methyl group [32]. A scale-up experiment was carried out in 3.0 g of (*S*)-**3** for the synthesis of (2*S*,8*S*)-**1** and near-gram scale product was obtained without decreasing the synthetic yield. All the analytic data of **1** are in accordance with those reported in the literature.

## 3. Materials and Methods

### 3.1. Chemistry

#### 3.1.1. General Method

All commercially available reagents were used without further purification. THF was distilled from sodium. CH_2_Cl_2_ was distilled from CaH_2_. Column chromatography was performed on silica gel (200–400 mesh). ^1^H-NMR and ^13^C-NMR spectra were recorded on a Bruker 500 MHz NMR spectrometer (Bruker, Fällanden, Switzerland). HRMS data were recorded on AB. SCIEX Triple TOF 5600+, LC-30AD (AB. SCIEX, Waltham, MA, USA).

#### 3.1.2. General Procedure for the Synthesis of Compounds

(*S*)-2-(2-Methylbutylthio)benzo[*d*]thiazole ((*S*)-**8**). To a stirred solution of (*S*)-**5** (0.500 g, 5.67 mmol) in THF (30.0 mL) at 0 °C was added Ph_3_P (1.785 g, 6.80 mmol) and **6** (1.138 g, 6.80 mmol). DIAD (1.375 g, 6.80 mmol) was added, and the resulting mixture was allowed to warm to room temperature and stirred for 4 h. After removal of solvent, the residue was purified by flash chromatography on silica gel (hexanes:ethyl acetate = 50:1) to afford compound (*S*)-**8** as a yellow oil (1.333 g, 99%). ${\left[\alpha \right]}_{D}^{20}$ = +22.0 (c = 0.5, CHCl_3_). ^1^H-NMR (500 MHz, CDCl_3_) δ 7.86 (d, *J* = 8.0 Hz, 1H), 7.74 (d, *J* = 7.5 Hz, 1H), 7.42–7.39 (m, 1H), 7.30–7.26 (m, 1H), 3.41 (dd, *J* = 6.0, 13.0 Hz, 1H), 3.21 (dd, *J* = 7.5, 13.0 Hz, 1H), 1.90–1.84 (m, 1H), 1.62–1.54 (m, 1H), 1.38–1.29 (m, 1H), 1.07 (d, *J* = 7.0 Hz, 3H), 0.96 (t, *J* = 7.5 Hz, 3H); ^13^C-NMR (125 MHz, CDCl_3_) δ 167.68, 153.32, 135.12, 125.92, 124.02, 121.38, 120.83, 40.32, 34.81, 28.69, 18.83, 11.27; HRMS (ESI) calcd for C_12_H_16_NS_2_^+^ [M + H]^+^: 238.0718, found: 238.0719.

HPLC analysis: Daicel Chiralcel OD-H column; hexane/*i*-propanol = 98:2, 0.7 mL/min, λ = 220 nm; t_R_ (major) = 9.31 min, t_R_ (minor) = 9.85 min; 99:1 *er*.

(*S*)-2-(2-Methylbutylsulfonyl)benzo[*d*]thiazole ((*S*)-**3**). To a solution of (*S*)-**8** (2.090 g, 8.80 mmol) in CH_2_Cl_2_ (90.0 mL) at room temperature was added *m*-CPBA (85% purity, 8.933 g, 44.00 mmol). Upon stirring overnight, the reaction mixture was quenched with saturated aqueous Na_2_S_2_O_3_ (10.0 mL) and NaHCO_3_ (20.0 mL), extracted with CH_2_Cl_2_, washed with brine, dried over anhydrous Na_2_SO_4_, concentrated, and purified by flash chromatography on silica gel (hexanes:ethyl acetate = 30:1) to give compound (*S*)-**3** as a pale yellow oil (2.229 g, 94%). ${\left[\alpha \right]}_{D}^{20}$ = +14.0 (c = 0.5, CHCl_3_). ^1^H-NMR (500 MHz, CDCl_3_) δ 8.21 (d, *J* = 8.0 Hz, 1H), δ 8.01 (d, *J* = 7.5 Hz, 1H), 7.65–7.57 (m, 2H), 3.55 (dd, *J* = 5.0, 14.5 Hz, 1H), 3.35 (dd, *J* = 8, 14.0 Hz, 1H), 2.28–2.19 (m, 1H), 1.59–1.51 (m, 1H), 1.43–1.34 (m, 1H), 1.13 (d, *J* = 7.0 Hz, 3H), 0.90 (t, *J* = 7.5 Hz, 3H); ^13^C-NMR (125 MHz, CDCl_3_) δ 166.80, 152.69, 136.72, 127.94, 127.59, 125.39, 122.33, 60.43, 29.92, 29.33, 19.30, 10.67; HRMS (ESI) calcd for C_12_H_15_NO_2_S_2_Na^+^ [M + Na]^+^: 292.0441, found: 292.0433.

(*R*)-4-Benzyl-3-butyryloxazolidin-2-one (**11**). To a stirred solution of **9** (10.000 g, 56.43 mmol) in THF (120.0 mL) at −78 °C under Ar was added *n*-BuLi (1.6 M in hexanes, 42.3 mL, 67.68 mmol) over 30 min. After stirred for 30 min, *n*-butyryl chloride (**10**) (8.6 mL, 84.70 mmol) was added dropwise. The reaction mixture was allowed to warmed to room temperature and stirred overnight, quenched with saturated aqueous NH_4_Cl, extracted with ethyl acetate, washed with brine, dried over anhydrous Na_2_SO_4_, concentrated, and purified by flash chromatography on silica gel (hexanes:ethyl acetate = 5:1) to give **11** as a colorless oil (13.950 g, 100%). ${\left[\alpha \right]}_{D}^{20}$ = −69.0 (c = 0.1, CHCl_3_). ^1^H-NMR (500 MHz, CDCl_3_) δ 7.35–7.21 (m, 5H), 4.69–4.66 (m, 1H), 4.21–4.15 (m, 2H), 3.31–3.28 (m, 1H), 2.99–2.85 (m, 2H), 2.77 (dd, *J* = 9.5, 13.0 Hz, 1H), 1.75–1.71 (m, 2H), 1.01 (t, *J* = 7.5 Hz, 3H); ^13^C-NMR (125 MHz, CDCl_3_) δ 173.17, 153.42, 135.29, 129.36, 128.88, 127.27, 66.10, 55.07, 37.88, 37.32, 17.65, 13.62. 

(*R*)-4-Benzyl-3-((*R*)-2-methylbutanoyl) oxazolidin-2-one (**12**). To a stirred solution of **11** (14.270 g, 57.70 mmol) in THF (280.0 mL) at −78 °C under Ar was added NaHMDS (2.0 M in THF, 58.0 mL, 116.00 mmol) dropwise. After 30 min, MeI (18.0 mL, 288.40 mmol) was added dropwise. Upon stirring at −78 °C for 2 h, the reaction was quenched with saturated aqueous NH_4_Cl, extracted with ethyl acetate, washed with brine, dried over anhydrous Na_2_SO_4_, concentrated, and purified by flash chromatography on silica gel (hexanes:ethyl acetate = 5:1) to give **12** as a pale yellow oil (12.034 g, 80%). ${\left[\alpha \right]}_{D}^{20}$ = −75.9 (c = 0.1, CHCl_3_). ^1^H-NMR (500 MHz, CDCl_3_) δ 7.34–7.21 (m, 5H), 4.70–4.67 (m, 1H), 4.21–4.16 (m, 2H), 3.66–3.62 (m, 1H), 3.29–3.26 (m, 1H), 2.77 (dd, *J* = 10.0, 13.5 Hz, 1H), 1.82–1.73 (m, 1H), 1.52–1.43 (m, 1H), 1.22 (d, *J* = 6.5 Hz, 3H), 0.93 (t, *J* = 7.0 Hz, 3H); ^13^C-NMR (125 MHz, CDCl_3_) δ 177.13, 153.05, 135.32, 129.41, 128.88, 127.28, 65.97, 55.30, 39.13, 37.87, 26.36, 16.85, 11.60.

(*R*)-2-Methylbutan-1-ol ((*R*)-**5**). To a solution of **12** (8.571 g, 32.8 mmol) in Et_2_O/MeOH (100.0 mL/3.0 mL) at −30 °C under Ar was added LiBH_4_ (2.0 M in THF, 11.5 mL, 23.0 mmol) dropwise. Upon stirring at −30 °C for 30 min, the reaction mixture was moved to an ice bath and stirred overnight, quenched with 10% aqueous NaOH, extracted with Et_2_O, dried over anhydrous Na_2_SO_4_, concentrated, and purified by flash chromatography on silica gel (*n*-pentane: Et_2_O = 5:1) to give (*R*)-**5** as a colorless oil (2.660 g, 92%). ${\left[\alpha \right]}_{D}^{20}$ = +5.0 (c = 1.0, CHCl_3_). ^1^H-NMR (500 MHz, CDCl_3_) δ 3.54–3.47 (m, 1H), 3.47–3.38 (m, 1H), 1.60–1.50 (m, 1H), 1.50–1.40 (m, 1H), 1.27–1.19 (m, 1H), 1.18–1.09 (m, 1H), 0.91 (t, *J* = 7.4 Hz, 3H), 0.91 (d, *J* = 6.7 Hz, 3H); ^13^C-NMR (500 MHz, CDCl_3_) δ 67.93, 37.31, 25.70, 16.04, 11.26.

(*R*)-2-(2-Methylbutylthio)benzo[*d*]thiazole ((*R*)-**8**). To a stirred solution of (*R*)-**5** (0.635 g, 7.20 mmol) in THF (70.0 mL) at 0 °C was added Ph_3_P (2.267 g, 8.64 mmol) and **6** (1.446 g, 8.64 mmol). DIAD (1.734 g, 8.64 mmol) was added, and the resulting mixture was allowed to warm to room temperature and stirred for 4 h. After removal of solvent, the residue was purified by flash chromatography on silica gel (hexanes:ethyl acetate = 50:1) to afford compound (*R*)-**8** as a yellow oil (1.400 g, 82%). ${\left[\alpha \right]}_{D}^{20}$ = −26.0 (c = 0.3, CHCl_3_). ^1^H-NMR (500 MHz, CDCl_3_) δ 7.86 (d, *J* = 8.0 Hz, 1H), 7.74 (d, *J* = 8.0 Hz, 1H), 7.40 (t, *J* = 8.0 Hz, 1H), 7.28 (dt, *J* = 1.0, 8.5 Hz, 1H), 3.41 (dd, *J* = 6.0, 13.0 Hz, 1H), 3.21 (dd, *J* = 7.5, 13.0 Hz, 1H), 1.90–1.84 (m, 1H), 1.62–1.54 (m, 1H), 1.38–1.29 (m, 1H), 1.07 (d, *J* = 6.5 Hz, 3H), 0.96 (t, *J* = 7.5 Hz, 3H); ^13^C-NMR (125 MHz, CDCl_3_) δ 167.73, 153.35, 135.16, 125.96, 124.06, 121.42, 120.87, 40.36, 34.84, 28.72, 18.86, 11.29.

HPLC analysis: Daicel Chiralcel OD-H column; hexane/*i*-propanol = 98:2, 0.7 mL/min, λ = 220 nm; t_R_ (major) = 9.79 min, t_R_ (minor) = 9.28 min; 93:7 *er*.

(*R*)-2-(2-Methylbutylsulfonyl)benzo[*d*]thiazole ((*R*)-**3**). To a solution of (*R*)-**8** (1.300 g, 5.48 mmol) in CH_2_Cl_2_ (55.0 mL) at room temperature was added *m*-CPBA (85% purity, 5.563 g, 27.40 mmol). Upon stirring overnight, the reaction mixture was quenched with saturated aqueous Na_2_S_2_O_3_ (10.0 mL) and NaHCO_3_ (20.0 mL), extracted with CH_2_Cl_2_, washed with brine, dried over anhydrous Na_2_SO_4_, concentrated, and purified by flash chromatography on silica gel (hexanes:ethyl acetate = 30:1) to give compound (*R*)-**3** as a pale yellow oil (1.357 g, 92%). ${\left[\alpha \right]}_{D}^{20}$ = −17.0 (c = 0.4, CHCl_3_). ^1^H-NMR (500 MHz, CDCl_3_) δ 8.20 (d, *J* = 8.0 Hz, 1H), δ 8.00 (d, *J* = 7.5 Hz, 1H), 7.65–7.57 (m, 2H), 3.55 (dd, *J* = 4.5, 14.0 Hz, 1H), 3.35 (dd, *J* = 8, 14.5 Hz, 1H), 2.26–2.20 (m, 1H), 1.58–1.51 (m, 1H), 1.43–1.34 (m, 1H), 1.13 (d, *J* = 6.5 Hz, 3H), 0.90 (t, *J* = 7.5 Hz, 3H); ^13^C-NMR (125 MHz, CDCl_3_) δ 166.81, 152.69, 136.73, 127.94, 127.59, 125.41, 122.33, 60.41, 29.93, 29.34, 19.31, 10.68.

(*S*)-6-(Benzyloxy)hex-4-yn-2-ol ((*S*)-**14**). To a stirred solution of **13** (5.350 g, 36.60 mmol) in THF (240.0 mL) at −78 °C under Ar was added *n*-BuLi (2.5 M in hexanes, 16.0 mL, 40.0 mmol). After 30 min, BF_3_^.^Et_2_O (5.0 mL, 39.90 mmol) was added, followed by (*S*)-2-methyloxirane ((*S*)-**7**) (1.933 g, 33.28 mmol). Upon stirring at −78 °C overnight, the reaction was quenched with saturated aqueous NH_4_Cl, extracted with ethyl acetate, washed with brine, dried over anhydrous Na_2_SO_4_, concentrated, and purified by flash chromatography on silica gel (hexanes:ethyl acetate = 10:1) to give (*S*)-**14** as a pale yellow oil (5.303 g, 78%). ${\left[\alpha \right]}_{D}^{20}$ = +8.0 (c = 0.2, CHCl_3_). ^1^H-NMR (500 MHz, CDCl_3_) 7.36–7.28 (m, 5H), 4.59 (s, 2H), 4.18 (d, *J* = 2.5 Hz, 2H), 3.99–3.93 (m, 1H), 2.48–2.36 (m, 2H), 2.06–1.85 (m, 1H), 1.27 (d, *J* = 6.0 Hz, 3H); ^13^C-NMR (125 MHz, CDCl_3_) δ 137.46, 128.41, 128.03, 127.83, 83.32, 78.51, 71.58, 66.33, 57.63, 29.36, 22.35.

(*S*)-6-(Benzyloxy)hex-4-yn-2-yl propionate ((*S*)-**15**). To a solution of (*S*)-**14** (5.000 g, 24.48 mmol) in CH_2_Cl_2_ (120.0 mL) at 0 °C was added propionic acid (2.720 g, 36.72 mmol), DCC (7.576 g, 36.72 mmol), and DMAP (0.300 g, 2.45 mmol). The reaction mixture was stirred overnight, diluted with water, washed with saturated aqueous NaHCO_3_, extracted with CH_2_Cl_2_, washed with brine, dried over anhydrous Na_2_SO_4_, concentrated, and purified by flash chromatography on silica gel (hexanes:ethyl acetate = 50:1) to give (*S*)-**15** as a pale yellow oil (6.245 g, 98%). ${\left[\alpha \right]}_{D}^{20}$ = −32.0 (c = 0.3, CHCl_3_). ^1^H-NMR (500 MHz, CDCl_3_) 7.36–7.28 (m, 5H), 5.05–4.99 (m, 1H), 4.58 (s, 2H), 4.16 (d, *J* = 2.0 Hz, 2H), 2.57–2.47 (m, 2H), 2.31 (q, *J* = 7.5 Hz, 2H), 1.34 (d, *J* = 6.5 Hz, 3H), 1.13 (t, *J* = 7.5 Hz, 3H); ^13^C-NMR (125 MHz, CDCl_3_) δ 173.82, 173.55, 128.40, 128.06, 127.80, 82.44, 78.06, 71.37, 68.52, 57.54, 27.78, 25.95, 19.23, 9.11; HRMS (ESI) calcd for C_16_H_20_NO_3_Na^+^ [M + Na]^+^: 283.1310, found: 283.1307.

(*S*)-6-Hydroxyhexan-2-yl propionate ((*S*)-**16**). To a stirred solution of (*S*)-**15** (1.200 g, 4.61 mmol) in MeOH (46.0 mL) was added Pd/C (10%, 0.200 g). The flask was evacuated and refilled with H_2_ (balloon). This process was repeated for 3 times. Upon stirring at room temperature for 24 h, the reaction mixture was filtered, concentrated, and purified by flash chromatography on silica gel (hexanes:ethyl acetate = 10:1) to give (*S*)-**16** as a colorless oil (0.747 g, 93%). ${\left[\alpha \right]}_{D}^{20}$ = +4.0 (c = 0.8, CHCl_3_). ^1^H-NMR (500 MHz, CDCl_3_) δ 4.94–4.88 (m, 1H), 3.63 (d, *J* = 6.5 Hz, 2H), 2.29 (q, *J* = 7.5 Hz, 2H), 1.64–1.35 (m, 7H), 1.20 (d, *J* = 6.5 Hz, 3H), 1.12 (t, *J* = 7.5 Hz, 3H); ^13^C-NMR (125 MHz, CDCl_3_) δ 174.22, 70.57, 62.70, 35.69, 32.47, 27.91, 21.64, 19.95, 9.18; HRMS (ESI) calcd for C_9_H_18_O_3_Na^+^ [M + Na]^+^: 197.1154, found: 197.1147.

(*S*)-6-Oxohexan-2-yl propionate ((*S*)-**4**). To a stirred solution of (*S*)-**16** (0.600 g, 3.44 mmol) in CH_2_Cl_2_ (35.0 mL) was added silica gel (1.100 g) and PCC (1.078 g, 5.0 mmol). Upon stirring at room temperature for 12 h, the reaction mixture was precipitated by adding petroleum ether, filtered through a pad of Celite, concentrated, and purified by flash chromatography on silica gel (hexanes:ethyl acetate = 50:1) to give (*S*)-**4** as a colorless oil (0.458 g, 77%). ${\left[\alpha \right]}_{D}^{20}$ = +5.3 (c = 0.8, CHCl_3_). ^1^H-NMR (500 MHz, CDCl_3_) δ 9.74 (t, *J* = 1.5 Hz, 1H), 4.93–4.87 (m, 1H), 2.46–2.42 (m, 2H), 2.28 (q, *J* = 7.5 Hz, 2H), 1.70–1.48 (m, 4H), 1.20 (d, *J* = 6.0 Hz, 3H), 1.11 (t, *J* = 7.5 Hz, 3H); ^13^C-NMR (125 MHz, CDCl_3_) δ 201.93, 174.06, 70.06, 43.45, 35.19, 27.84, 19.86, 17.88, 9.12; HRMS (ESI) calcd for C_18_H_32_O_6_Na^+^ [2M + Na]^+^: 367.2096, found: 367.2082.

(*R*)-6-(Benzyloxy)hex-4-yn-2-yl propionate ((*R*)-**14**). To a stirred solution of **13** (6.493 g, 44.41 mmol) in THF (250.0 mL) at −78 °C under Ar was added *n*-BuLi (2.5 M in hexanes, 21.3 mL, 53.25 mmol). After 30 min, BF_3_^.^Et_2_O (6.7 mL, 53.47 mmol) was added, followed by (*R*)-2-methyloxirane ((*R*)-**7**) (2.580 g, 44.41 mmol). Upon stirring at −78 °C overnight, the reaction was quenched with saturated aqueous NH_4_Cl, extracted with ethyl acetate, washed with brine, dried over anhydrous Na_2_SO_4_, concentrated, and purified by flash chromatography on silica gel (hexanes:ethyl acetate = 30:1) to give (*R*)-**14** as a pale yellow oil (6.895 g, 76%). ${\left[\alpha \right]}_{D}^{20}$ = −13.2 (c = 0.1, CHCl_3_).

(*R*)-6-(Benzyloxy)hex-4-yn-2-yl propionate ((*R*)-**15**). To a solution of (*R*)-**14** (6.000 g, 29.37 mmol) in CH_2_Cl_2_ (150.0 mL) at 0 °C was added propionic acid (3.264 g, 44.06 mmol), DCC (9.091 g, 44.06 mmol), and DMAP (0.359 g, 2.94 mmol). The reaction mixture was stirred overnight, diluted with water, washed with saturated aqueous NaHCO_3_, extracted with CH_2_Cl_2_, washed with brine, dried over anhydrous Na_2_SO_4_, concentrated, and purified by flash chromatography on silica gel (hexanes:ethyl acetate = 50:1) to give (*R*)-**15** as a pale yellow oil (7.647 g, 100%). ${\left[\alpha \right]}_{D}^{20}$ = +33.8 (c = 0.1, CHCl_3_).

(*R*)-6-Hydroxyhexan-2-yl propionate ((*R*)-**16**). To a stirred solution of (*R*)-**15** (4.100 g, 15.75 mmol) in MeOH (158.0 mL) was added Pd/C (10%, 1.300 g). The flask was evacuated and refilled with H_2_ (balloon). This process was repeated for 3 times. Upon stirring at room temperature for 24 h, the reaction mixture was filtered, concentrated, and purified by flash chromatography on silica gel (hexanes:ethyl acetate = 10:1) to give (*R*)-**16** as a colorless oil (2.333 g, 85%). ${\left[\alpha \right]}_{D}^{20}$ = −8.4 (c = 0.1, CHCl_3_). 

(*R*)-6-Oxohexan-2-yl propionate ((*R*)-**4**). To a stirred solution of (*R*)-**16** (1.900 g, 10.90 mmol) in CH_2_Cl_2_ (100.0 mL) was added silica gel (3.500 g) and PCC (3.400 g, 16.77 mmol). Upon stirring at room temperature for 12 h, the reaction mixture was precipitated by adding petroleum ether, filtered through a pad of Celite, concentrated, and purified by flash chromatography on silica gel (hexanes:ethyl acetate = 50:1) to give (*R*)-**4** as a colorless oil (1.478 g, 79%). ${\left[\alpha \right]}_{D}^{20}$ = −6.1 (c = 0.9, CHCl_3_).

General procedure for the preparation of 8-methyldec-6-en-2-yl propionate (1). To a stirred solution of sulfone **3** (1.0 equiv.) in THF (0.1 M) at −78 °C under Ar was added NaHMDS (2.0 M in THF, 1.2 equiv.). After 30 min, a solution of aldehyde **4** (1.2 equiv.) in THF (1.0 M) was added dropwise. The reaction mixture was slowly warmed to −50 °C and stirred overnight, quenched with saturated aqueous NH_4_Cl, extracted with ethyl acetate, washed with brine, dried over anhydrous Na_2_SO_4_, concentrated, and purified by flash chromatography on silica gel (hexanes:ethyl acetate = 100:1) to give **2** as a colorless oil.

To a stirred solution of **2** (1.0 equiv.) in EtOH (0.1 M) was added Pt/C (10%, 8.3% Pt). The flask was evacuated, and then filled with H_2_ (balloon). This process was repeated for 3 times. After 24 h, the reaction mixture was filtered, concentrated, and purified by flash chromatography on silica gel (hexanes:ethyl acetate = 100:1) to give **1** as a colorless oil.

(2*S*,8*S*)-8-Methyldecan-2-yl propionate ((2*S*,8*S*)-**1**). Sulfone (*S*)-**3** (0.627 g, 2.33 mmol) and aldehyde (*S*)-**4** (0.481 g, 2.79 mmol) were used to give: (2*S*,8*S*)-**2** as a colorless oil (0.293 g, 56%), ${\left[\alpha \right]}_{D}^{20}$ = +9.7 (c = 0.4, CHCl_3_), *Z*/*E*: 2/3 mixture, major: ^1^H-NMR (500 MHz, CDCl_3_) δ 5.35–5.22 (m, 1H), 5.15–5.08 (m, 1H), 4.95–4.86 (m, 1H), 2.36–2.24 (m, 3H), 2.07–1.92 (m, 2H), 1.62–1.22 (m, 6H), 1.20 (d, *J* = 6.3 Hz, 3H), 1.13 (t, *J* = 7.6 Hz, 3H), 0.91 (d, *J* = 6.7 Hz, 3H), 0.83 (t, *J* = 7.4 Hz, 3H); ^13^C-NMR (125 MHz, CDCl_3_) δ 174.15, 136.66, 127.86, 70.61, 35.55, 33.39, 30.21, 27.94, 27.18, 25.65, 21.00, 20.00, 11.91, 9.20; (2*S*,8*S*)-**1** as a colorless oil (0.271 g, 92%), ${\left[\alpha \right]}_{D}^{20}$ = +6.8 (c = 0.7, CHCl_3_), ^1^H-NMR (500 MHz, CDCl_3_) δ 4.92–4.86 (m, 1H), 2.29 (q, *J* = 7.6 Hz, 2H), 1.60–1.52 (m, 1H), 1.50–1.41 (m, 1H), 1.36–1.21 (m, 9H), 1.19 (d, *J* = 6.5 Hz, 3H), 1.13 (t, *J* = 7.6 Hz, 3H), 1.16–1.03 (m, 2H), 0.84 (t, *J* = 7.2 Hz, 3H), 0.83 (d, *J* = 6.3 Hz, 3H); ^13^C-NMR (125 MHz, CDCl_3_) δ 174.19, 70.81, 36.49, 35.96, 34.35, 29.78, 29.46, 27.95, 26.96, 25.43, 19.98, 19.18, 11.38, 9.22; HRMS (ESI) calcd for C_14_H_28_O_2_Na^+^ [M + Na]^+^: 251.1987, found: 251.1979.

(2*R*,8*S*)-8-Methyldecan-2-yl propionate ((2*R*,8*S*)-**1**). Sulfone (*S*)-**3** (0.216 g, 0.80 mmol) and aldehyde (*R*)-**4** (0.166 g, 0.96 mmol) were used to give: (2*R*,8*S*)-**2** as a colorless oil (0.114 g, 63%), ${\left[\alpha \right]}_{D}^{20}$ = +11.0 (c = 0.2, CHCl_3_), *Z*/*E*: 4/5 mixture, major: ^1^H-NMR (500 MHz, CDCl_3_) δ 5.35–5.21 (m, 2H), 4.95–4.86 (m, 1H), 2.34-2.25 (m, 3H), 2.07–1.90 (m, 2H), 1.61–1.23 (m, 6H), 1.19 (d, *J* = 6.3 Hz, 3H), 1.12 (t, *J* = 7.6 Hz, 3H), 0.91 (d, *J* = 6.7 Hz, 3H), 0.83 (t, *J* = 7.5 Hz, 3H); ^13^C-NMR (125 MHz, CDCl_3_) δ 174.66, 137.15, 128.36, 71.10, 36.03, 33.88, 30.71, 28.42, 27.66, 26.16, 21.51, 20.49, 12.44, 9.70; (2*R*,8*S*)-**1** as a colorless oil (0.095 g, 83%), ${\left[\alpha \right]}_{D}^{20}$ = +2.3 (c = 0.6, CHCl_3_), ^1^H-NMR (500 MHz, CDCl_3_) δ 4.92–4.86 (m, 1H), 2.28 (q, *J* = 7.6 Hz, 2H), 1.57–1.53 (m, 1H), 1.48–1.42 (m, 1H), 1.28–1.25 (m, 9H), 1.19 (d, *J* = 6.5 Hz, 3H), 1.12 (t, *J* = 7.6 Hz, 3H), 1.16–1.01 (m, 2H), 0.84 (t, *J* = 7.5 Hz, 3H), 0.83 (d, *J* = 6.0 Hz, 3H); ^13^C-NMR (125 MHz, CDCl_3_) δ 174.13, 70.79, 36.49, 35.96, 34.35, 29.78, 29.45, 27.94, 26.95, 25.42, 19.97, 19.17, 11.36, 9.20; HRMS (ESI) calcd for C_14_H_28_O_2_Na^+^ [M + Na]^+^: 251.1987, found: 251.1985. 

(2*S*,8*R*)-8-Methyldecan-2-yl propionate ((2*S*,8*R*)-**1**). Sulfone (*R*)-**3** (0.563 g, 2.09 mmol) and aldehyde (*S*)-**4** (0.432 g, 2.51 mmol) were used to give: (2*S*,8*R*)-**2** as a colorless oil (0.280 g, 59%), ${\left[\alpha \right]}_{D}^{20}$ = −10.4 (c = 0.7, CHCl_3_), *Z*/*E*: 1/2 mixture, major: ^1^H-NMR (500 MHz, CDCl_3_) δ 5.36–5.06 (m, 2H), 4.96–4.84 (m, 1H), 2.35–2.23 (m, 3H), 2.09–1.89 (m, 2H), 1.65–1.21 (m, 6H), 1.19 (d, *J* = 6.2 Hz, 3H), 1.12 (t, *J* = 7.6 Hz, 3H), 0.91 (d, *J* = 6.7 Hz, 3H), 0.83 (t, *J* = 7.4 Hz, 3H); ^13^C-NMR (125 MHz, CDCl_3_) δ 174.14, 136.63, 127.85, 70.58, 35.52, 33.37, 30.20, 27.91, 27.14, 25.66, 21.01, 19.98, 11.93, 9.19; (2*S*,8*R*)-**1** as a colorless oil (0.248 g, 88%), ${\left[\alpha \right]}_{D}^{20}$ = −2.3 (c = 0.6, CHCl_3_), ^1^H-NMR (500 MHz, CDCl_3_) δ 4.94–4.84 (m, 1H), 2.28 (q, *J* = 7.6 Hz, 2H), 1.63–1.51 (m, 1H), 1.50–1.39 (m, 1H), 1.37–1.20 (m, 9H), 1.19 (d, *J* = 6.2 Hz, 3H), 1.12 (t, *J* = 7.6 Hz, 3H), 1.15–1.00 (m, 2H), 0.84 (t, *J* = 7.1 Hz, 3H), 0.83 (d, *J* = 5.3 Hz, 3H); ^13^C-NMR (125 MHz, CDCl_3_) δ 174.16, 70.79, 36.48, 35.95, 34.33, 29.78, 29.45, 27.93, 26.95, 25.43, 19.97, 19.17, 11.37, 9.21. 

(2*R*,8*R*)-8-Methyldecan-2-yl propionate ((2*R*,8*R*)-**1**). Sulfone (*R*)-**3** (0.453 g, 1.68 mmol) and aldehyde (*R*)-**4** (0.346 g, 2.01 mmol) were used to give: (2*R*,8*R*)-**2** as a colorless oil (0.236 g, 62%), ${\left[\alpha \right]}_{D}^{20}$ = −9.1 (c = 0.6, CHCl_3_), *Z*/*E*: 2/3 mixture, major: ^1^H-NMR (500 MHz, CDCl_3_) δ 5.36–5.07 (m, 2H), 4.95–4.86 (m, 1H), 2.36–2.24 (m, 3H), 2.07–1.91 (m, 2H), 1.65–1.23 (m, 6H), 1.20 (d, *J* = 6.3 Hz, 3H), 1.13 (t, *J* = 7.6 Hz, 3H), 0.92 (d, *J* = 6.7 Hz, 3H), 0.83 (t, *J* = 7.4 Hz, 3H); ^13^C-NMR (125 MHz, CDCl_3_) δ 174.13, 136.66, 127.87, 70.60, 35.56, 33.39, 30.22, 27.94, 27.19, 25.66, 21.00, 20.00, 11.91, 9.20; (2*R*,8*S*)-**1** as a colorless oil (0.186 g, 78%), ${\left[\alpha \right]}_{D}^{20}$ = −8.6 (c = 0.3, CHCl_3_), ^1^H-NMR (500 MHz, CDCl_3_) δ 4.93–4.84 (m, 1H), 2.28 (q, *J* = 7.6 Hz, 2H), 1.62–1.51 (m, 1H), 1.50–1.39 (m, 1H), 1.36–1.20 (m, 9H), 1.19 (d, *J* = 6.3 Hz, 3H), 1.12 (t, *J* = 7.6 Hz, 3H), 1.15–1.02 (m, 2H), 0.84 (t, *J* = 7.4 Hz, 3H), 0.82 (d, *J* = 6.2 Hz, 3H); ^13^C-NMR (125 MHz, CDCl_3_) δ 174.16, 70.79, 36.48, 35.95, 34.33, 29.77, 29.44, 27.93, 26.95, 25.42, 19.97, 19.16, 11.36, 9.20.

## 4. Conclusions

We describe a convergent asymmetric synthesis of four stereoisomers of **1**, a sex pheromone of the western corn rootworm. The molecular skeleton was connected between chiral BT-sulfone and chiral aldehyde through a key Julia–Kocienski coupling. This synthetic route provided the four stereoisomers of **1** in 24–29% total yield via a six-step sequence. The simple, convenient, and efficient asymmetric synthetic route to **1** will be highly helpful for the further practical testing and use of pheromones as benign environmental tools for pest control.

## Supplementary Materials

The following are available online http://www.mdpi.com/1420-3049/23/3/667/s1. HPLC data for compounds *S*-**8** and *R*-**8**; NMR spectra for all synthetic compounds.
