# Supplementary material for: Asymmetric Total Synthesis of Four Stereoisomers of the Sex Pheromone of the Western Corn Rootworm
Source: Molecules. 2018 Mar 15;23(3):667. doi: 10.3390/molecules23030667 (PMC6017750; doi:10.3390/molecules23030667)
Supplement: Supplementary file 1 [file molecules-23-00667-s001.pdf]

Supporting Information

Asymmetric total synthesis of four stereoisomers of the sex pheromone of the western corn rootworm

Zhi-Feng Sun [1,2,†], Tao Zhang [1,†], Jinyang Liu [1], Zhen-Ting Du [1,3,*], and Huaiji Zheng [1,3,*]

[1] *Shaanxi Key Laboratory of Natural Products and Chemical Biology, College of Chemistry and Pharmacy, North West Agriculture and Forestry University, Yangling 712100, China*
[2] *Shaanxi Key Laboratory for Catalysis, College of Chemical and Environment Science, Shaanxi University of Technology, Hanzhong 723001, China*
[3] *Key Laboratory of Botanical Pesticide R&D in Shaanxi Province, Yangling 712100, China*
† *These Authors contributed equally to this work.*

hjzheng@nwsuaf.edu.cn (H. Zheng); duzt@nwsuaf.edu.cn (Z.-T. Du)

(*S*)-2-(2-Methylbutylthio)benzo[*d*]thiazole ((*S*)-8).

HPLC analysis: Daicel Chiralcel OD-H column; hexane/*i*-propanol = 98:2, 0.7 mL/min, λ = 220 nm; t_R (major) = 9.31 min, t_R (minor) = 9.85 min. 99:1 *er*.

HPLC Report

Sample Name:SZF-1118-+- OD-H 220 982 0.7 Recording Time:2017.11.18 20:53

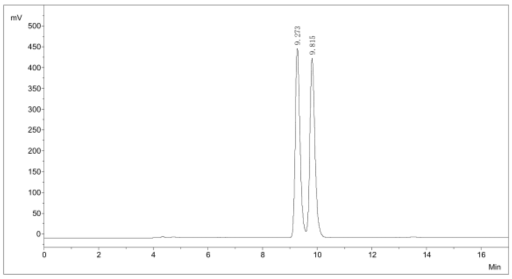

No.	PeakNo	ID.Name	R.Time	PeakHeight	PeakArea	Conc
1	1		9.273	454267.9	5533465.7	49.6543
2	2		9.815	431099.0	5610506.6	50.3457
Total				885366.9	11143972.3	100.0000

No.	IDName	Mi(ug)	MO(ug)	Cm(ug/m3)	Cc(mg/m3)

HPLC Report

Sample Name:SZF-1118-S OD-H 220 982 0.7 Recording Time:2017.11.18 21:11

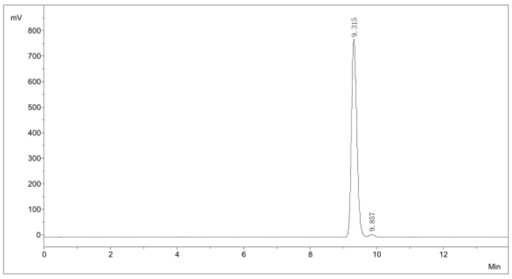

No.	PeakNo	ID.Name	R.Time	PeakHeight	PeakArea	Conc
1	1		9.315	771833.1	8632533.9	99.0011
2	2		9.857	8495.7	87099.4	0.9989
Total				780328.8	8719633.3	100.0000

No.	IDName	Mi(ug)	MO(ug)	Cm(ug/m3)	Cc(mg/m3)

(*R*)-2-(2-methylbutylthio)benzo[*d*]thiazole ((*R*)-8).

HPLC analysis: Daicel Chiralcel OD-H column; hexane/*i*-propanol = 98:2, 0.7 mL/min, λ = 220 nm; t_R (major) = 9.79 min, t_R (minor) = 9.28 min. 93:7 *er*.

HPLC Report

Sample Name:SZF-1118-+- OD-H 220 982 0.7 Recording Time:2017.11.18 20:53

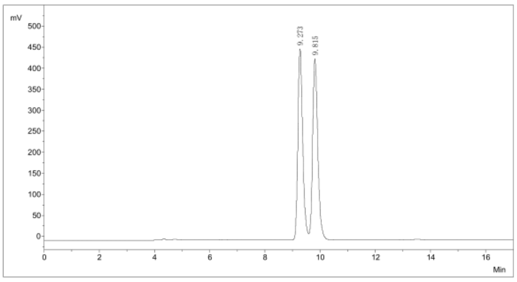

No.	PeakNo	ID.Name	R.Time	PeakHeight	PeakArea	Conc
1	1		9.273	454267.9	5533465.7	49.6543
2	2		9.815	431099.0	5610506.6	50.3457
Total				885366.9	11143972.3	100.0000

No.	IDName	Mi(ug)	MO(ug)	Cm(ug/m3)	Cc(mg/m3)

HPLC Report

Sample Name:SZF-1118-R OD-H 220 982 0.7 Recording Time:2017.11.18 21:26

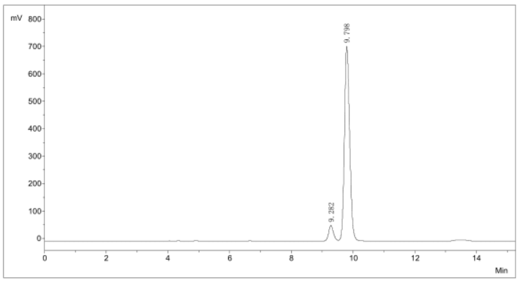

No.	PeakNo	ID.Name	R.Time	PeakHeight	PeakArea	Conc
1	1		9.282	56541.6	625749.9	6.9517
2	2		9.798	709855.4	8375667.5	93.0483
Total				766397.1	9001417.4	100.0000

No.	IDName	Mi(ug)	MO(ug)	Cm(ug/m3)	Cc(mg/m3)

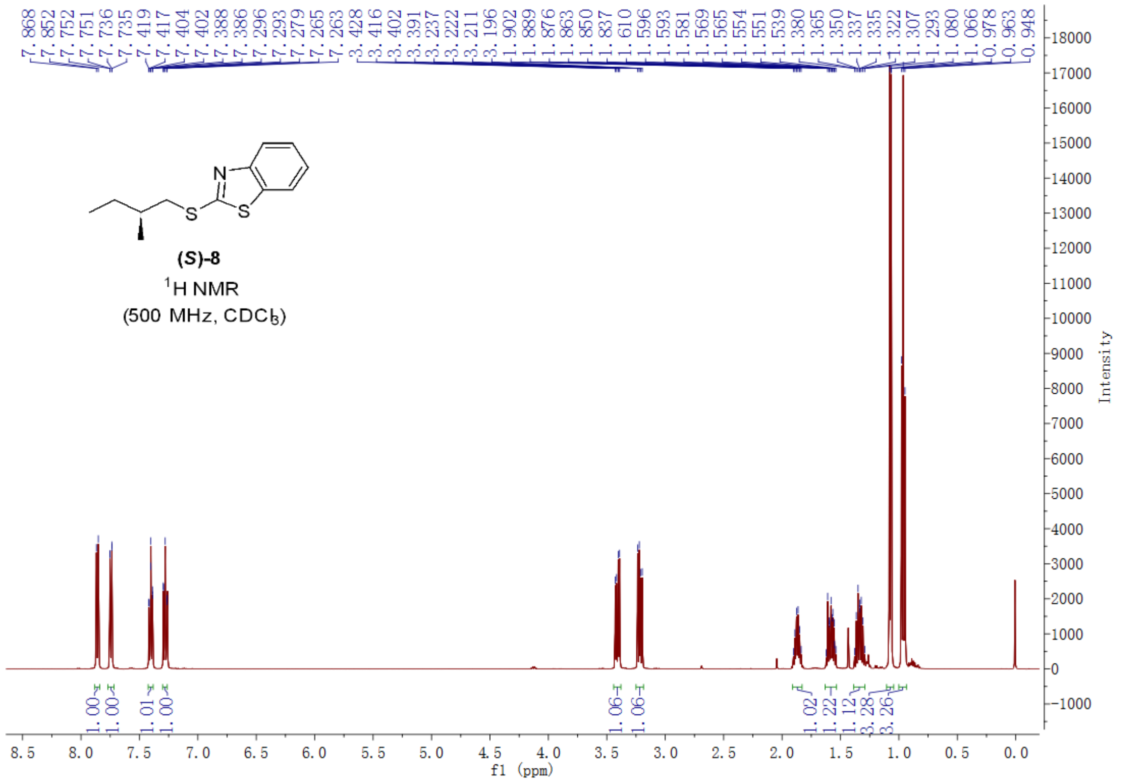
(S)-8
^{1}H NMR
(500 MHz, CDCl$_3$)
f1 (ppm)
Intensity

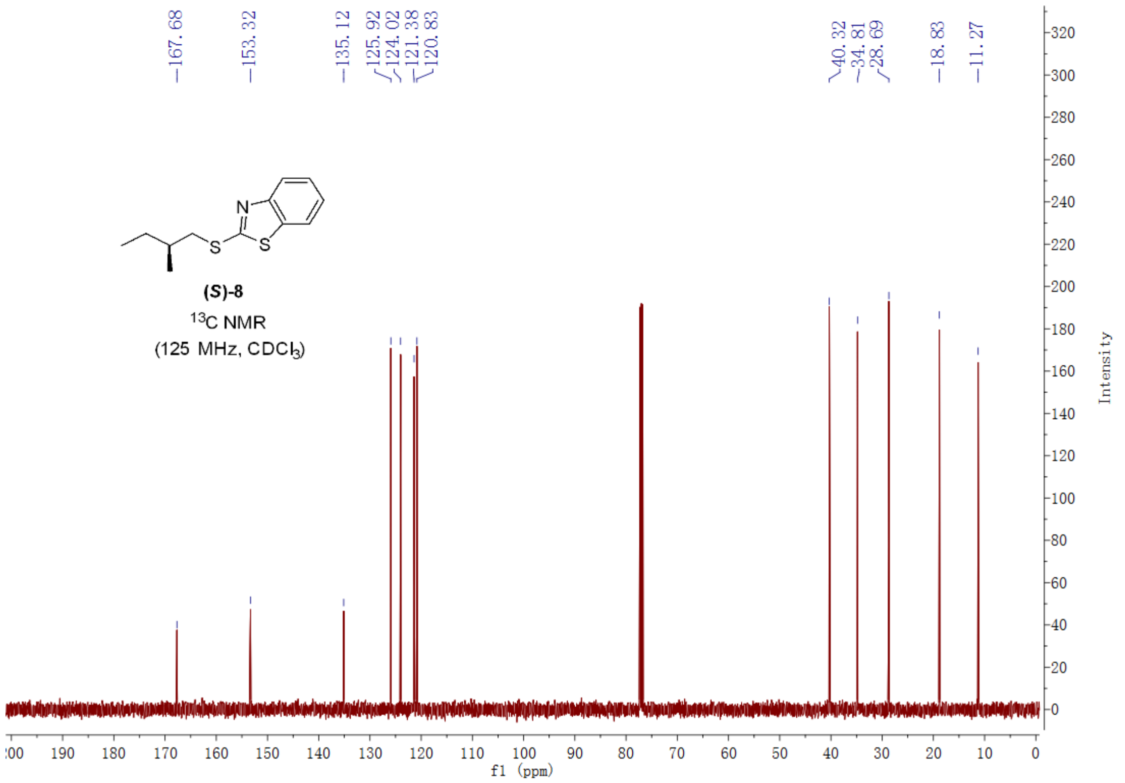
(S)-8
^{13}C NMR
(125 MHz, CDCl$_3$)
f1 (ppm)
Intensity

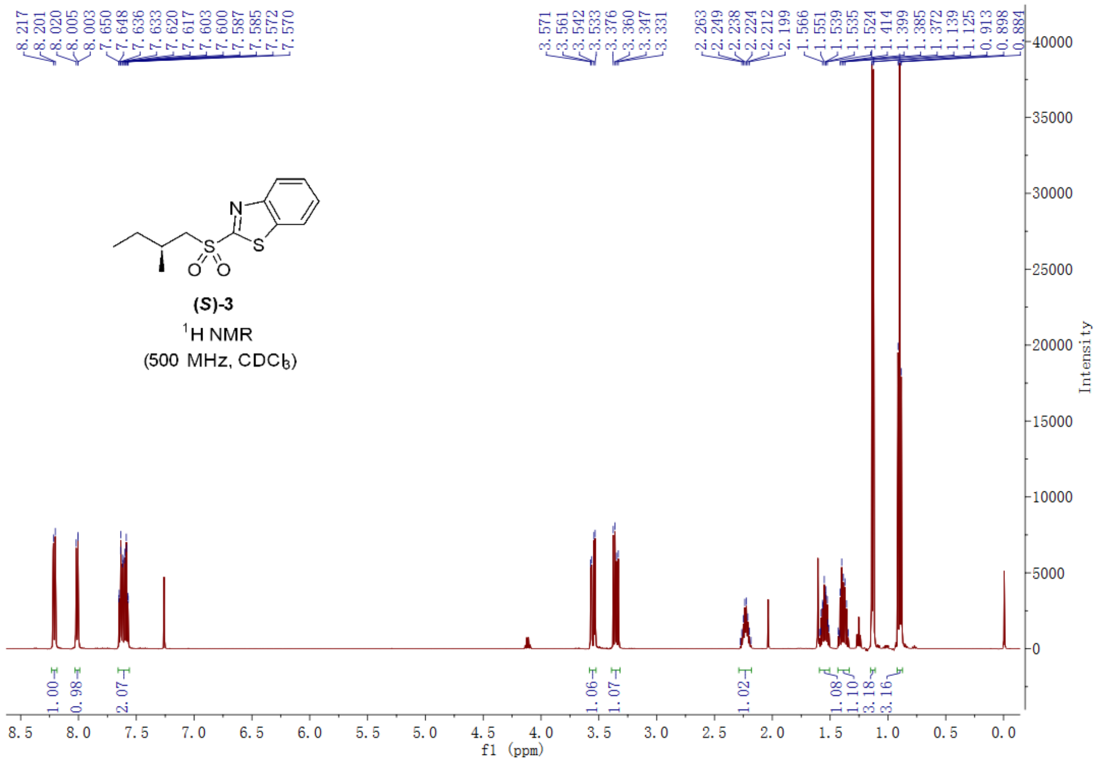

(*S*)-3

^{1}H NMR

(500 MHz, $CDCl_3$)

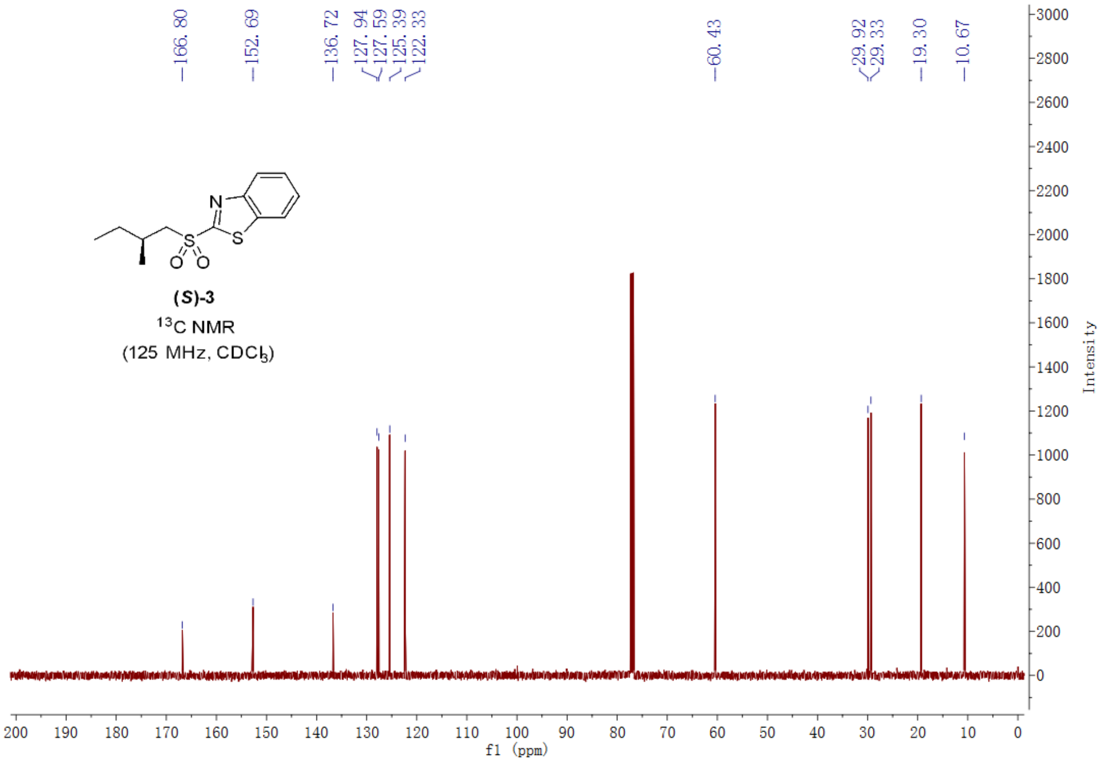

(*S*)-3

^{13}C NMR

(125 MHz, $CDCl_3$)

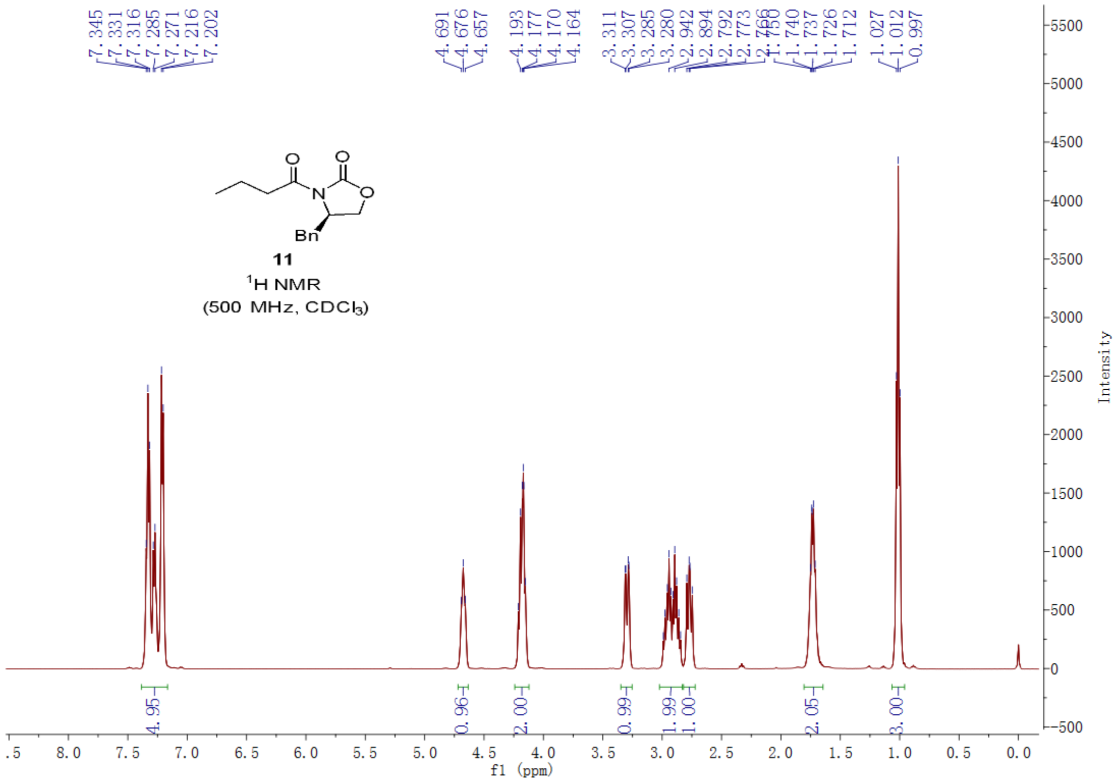
7.345
7.331
7.316
7.285
7.271
7.216
7.202
4.691
4.676
4.657
4.193
4.177
4.170
4.164
3.311
3.307
3.285
3.280
2.942
2.894
2.792
2.773
1.740
1.737
1.726
1.712
1.027
1.012
0.997
Bn
11
1H NMR
(500 MHz, CDCl3)
4.95
0.96
2.00
0.99
1.99
1.00
2.05
3.00
f1 (ppm)
Intensity

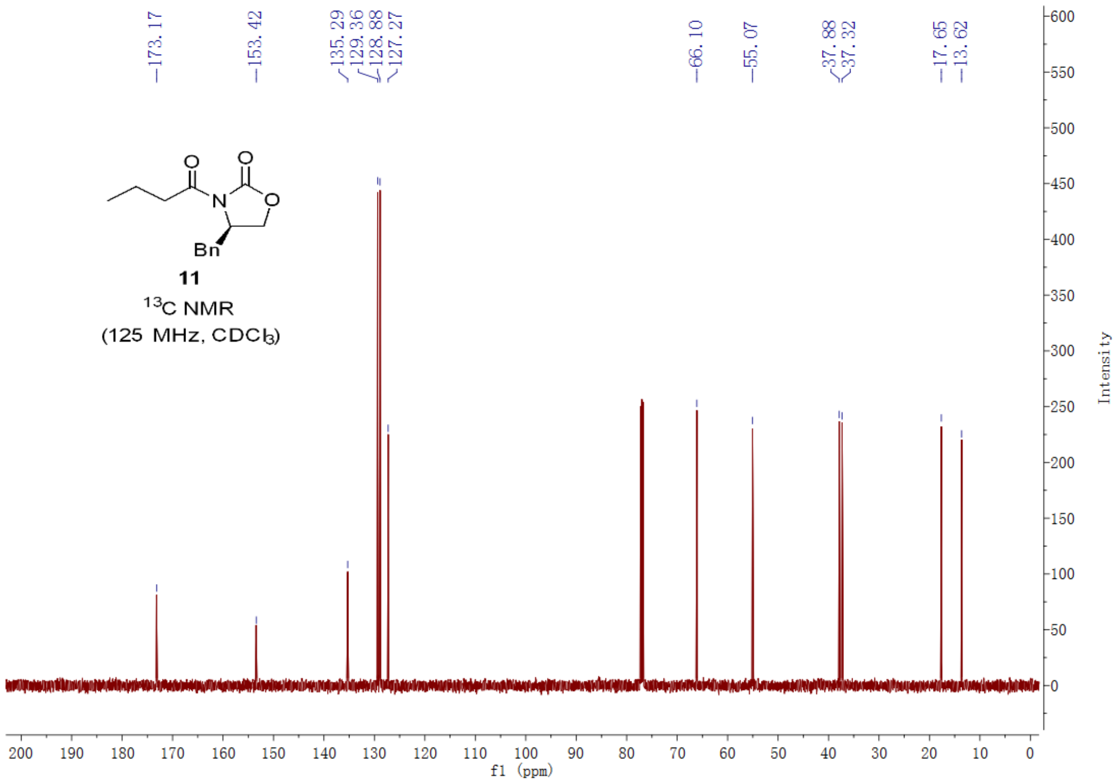
173.17
153.42
135.29
129.36
128.88
127.27
66.10
55.07
37.88
37.32
17.65
13.62
Bn
11
13C NMR
(125 MHz, CDCl3)
f1 (ppm)
Intensity

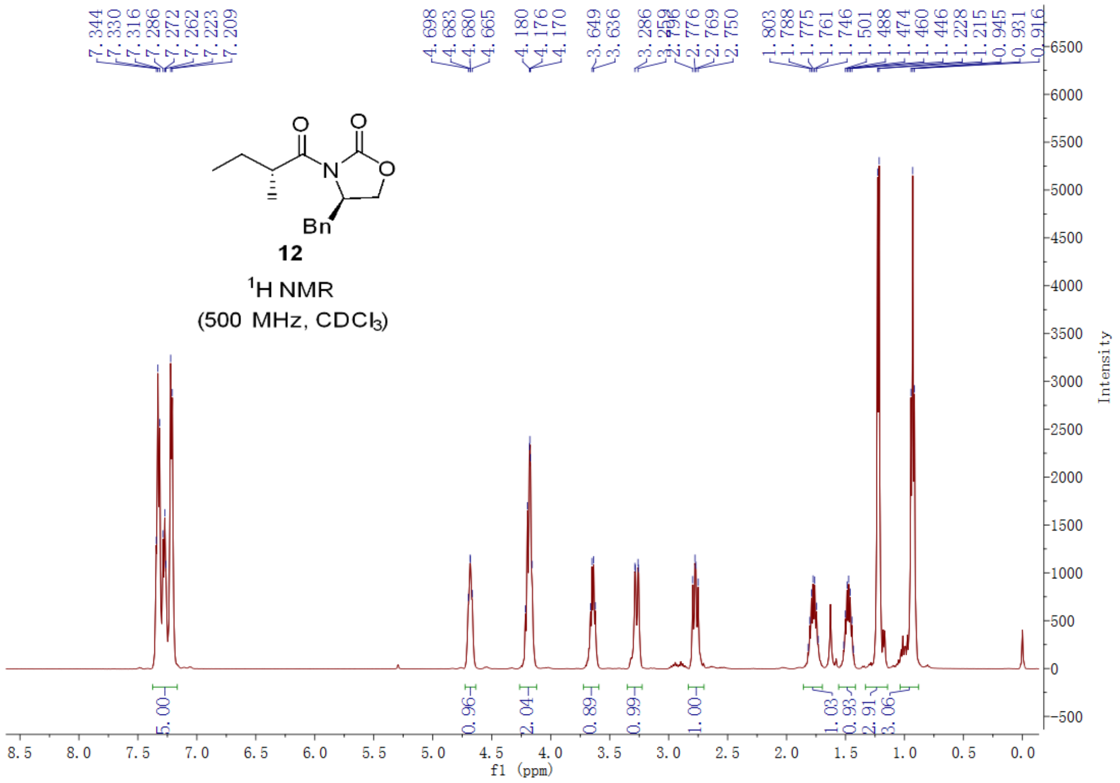
12
^{1}H NMR
(500 MHz, CDCl3)
f1 (ppm)
Intensity

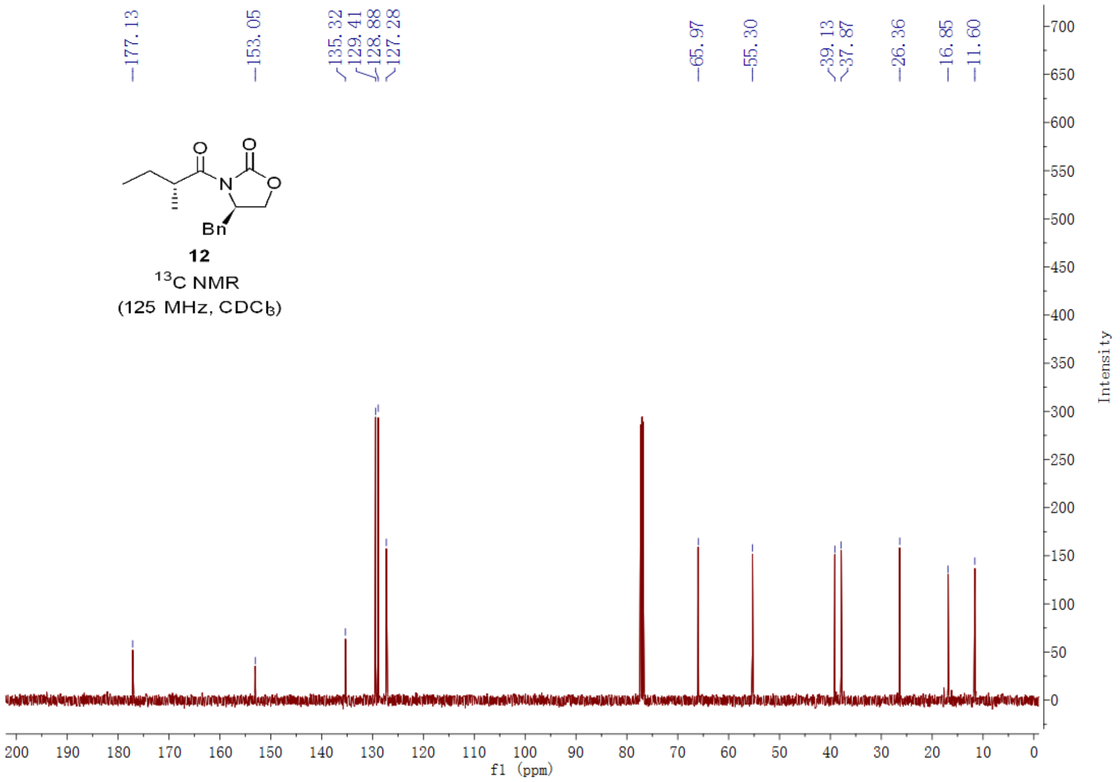
12
^{13}C NMR
(125 MHz, CDCl3)
f1 (ppm)
Intensity

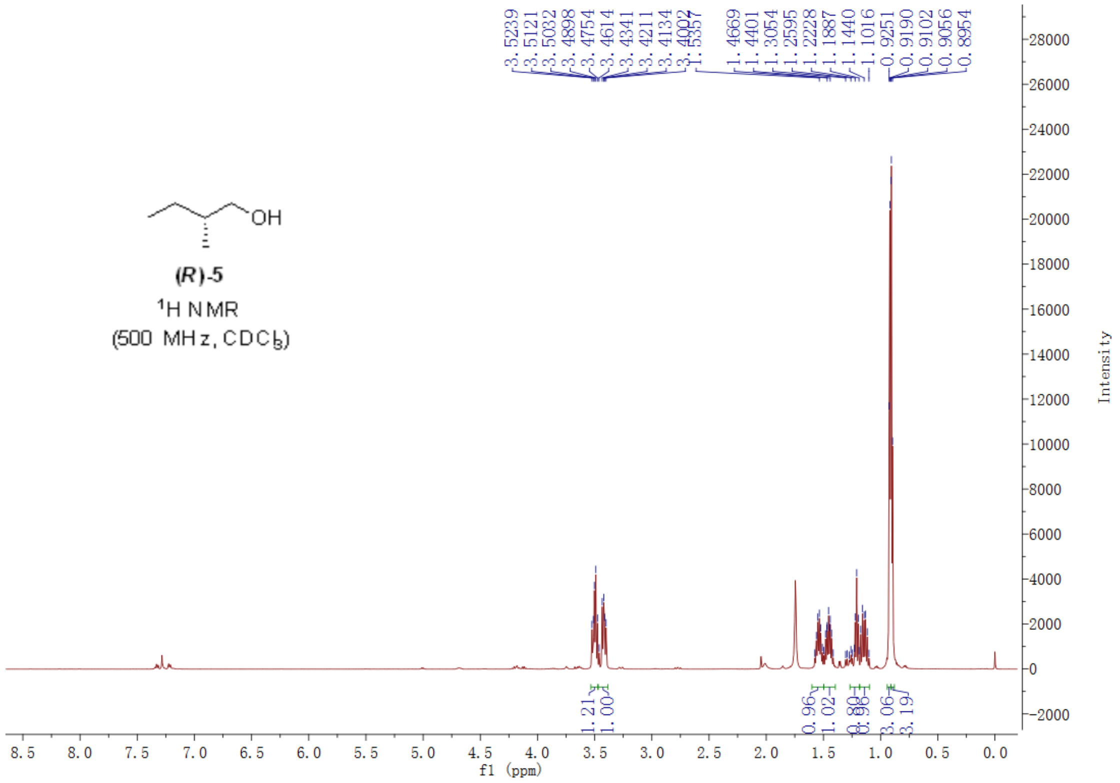
OH
(R)-5
1H NMR
(500 MHz, CDCl3)
f1 (ppm)
Intensity

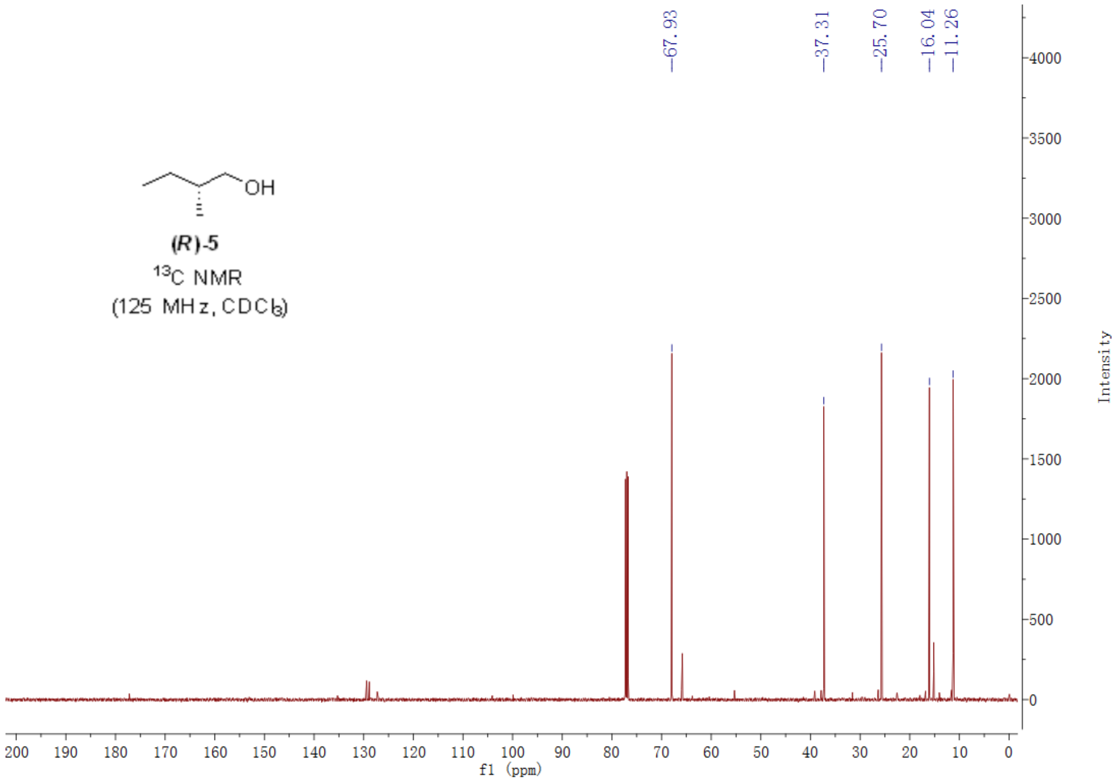
OH
(R)-5
13C NMR
(125 MHz, CDCl3)
67.93
37.31
25.70
16.04
11.26
f1 (ppm)
Intensity

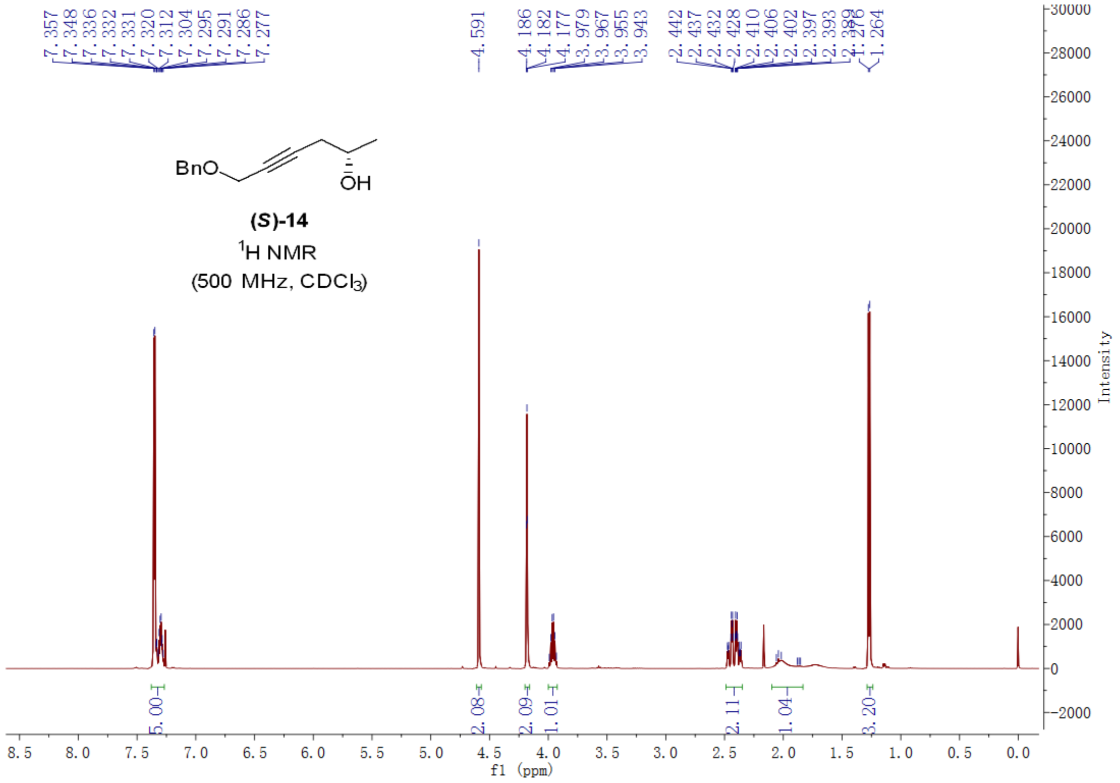
BnO
OH
(S)-14
1H NMR
(500 MHz, CDCl3)
Intensity
f1 (ppm)

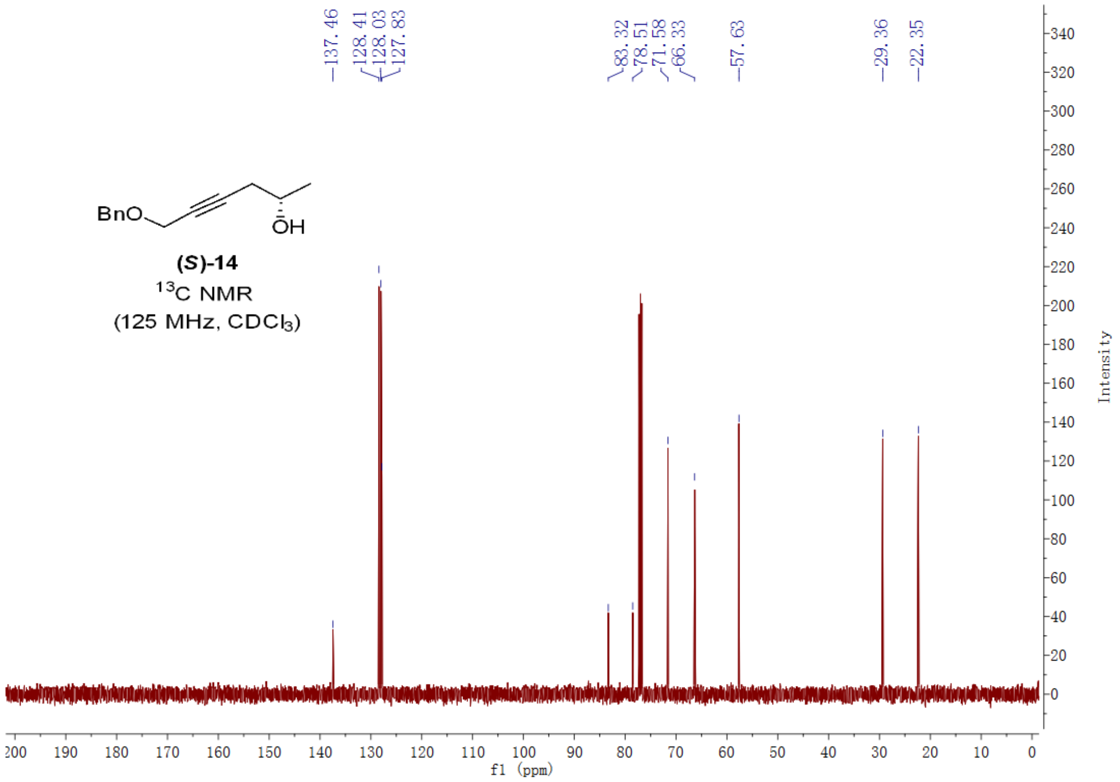
BnO
OH
(S)-14
13C NMR
(125 MHz, CDCl3)
Intensity
f1 (ppm)

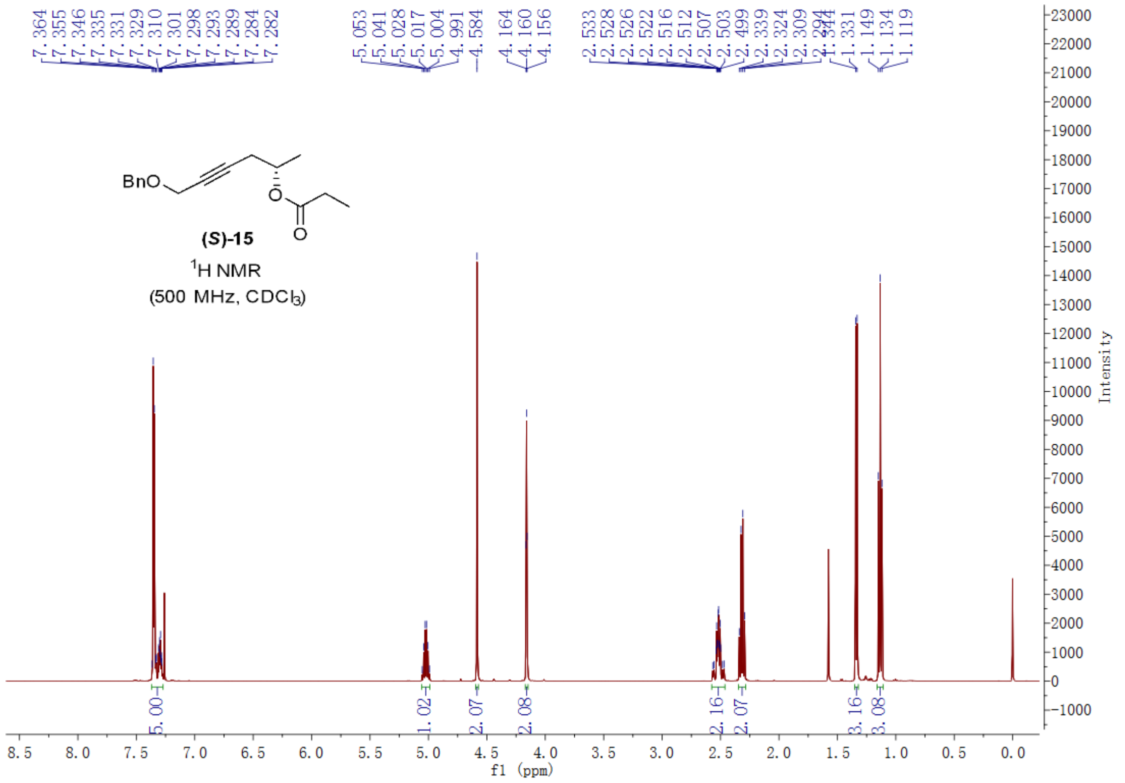
BnO
(S)-15
^{1}H NMR
(500 MHz, $CDCl_3$)
7.364
7.355
7.346
7.335
7.331
7.329
7.310
7.301
7.298
7.293
7.289
7.284
7.282
5.053
5.041
5.028
5.017
5.004
4.991
4.584
4.164
4.160
4.156
2.533
2.528
2.526
2.522
2.516
2.512
2.507
2.503
2.499
2.339
2.324
2.309
2.294
1.344
1.331
1.149
1.134
1.119
5.00
1.02
2.07
2.08
2.16
2.07
3.16
3.08
f1 (ppm)
Intensity

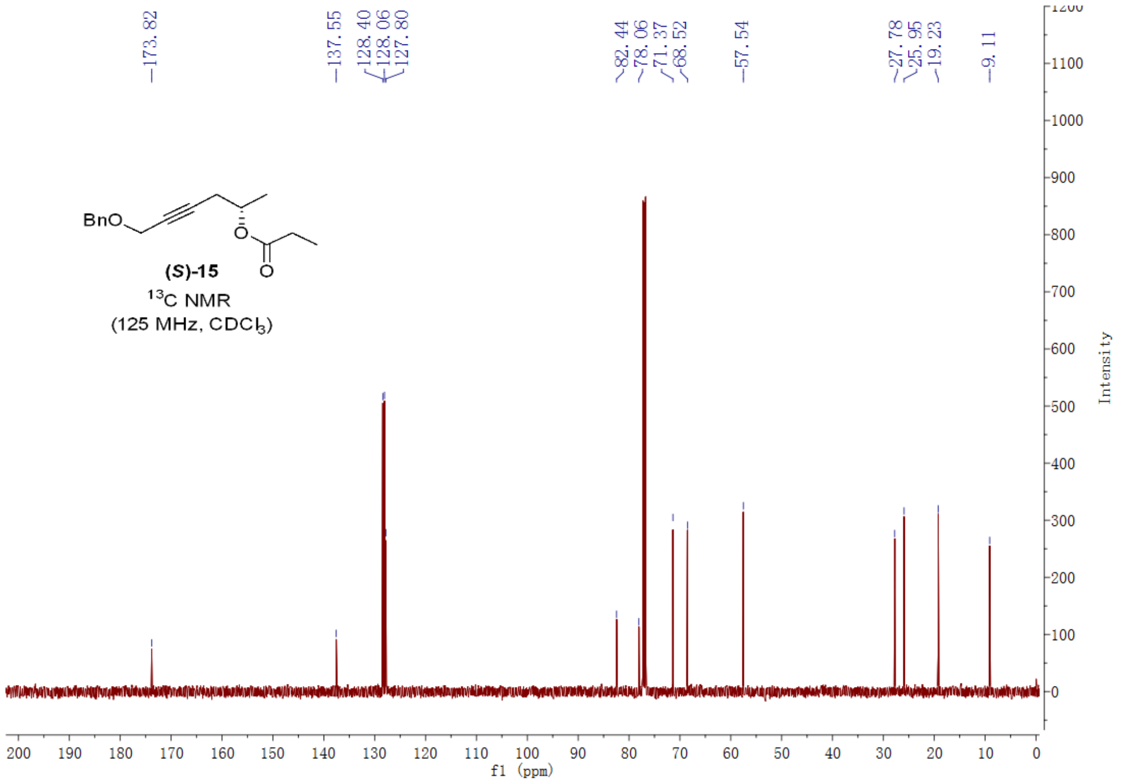
BnO
(S)-15
^{13}C NMR
(125 MHz, $CDCl_3$)
173.82
137.55
128.40
128.06
127.80
82.44
78.06
71.37
68.52
57.54
27.78
25.95
19.23
9.11
f1 (ppm)
Intensity

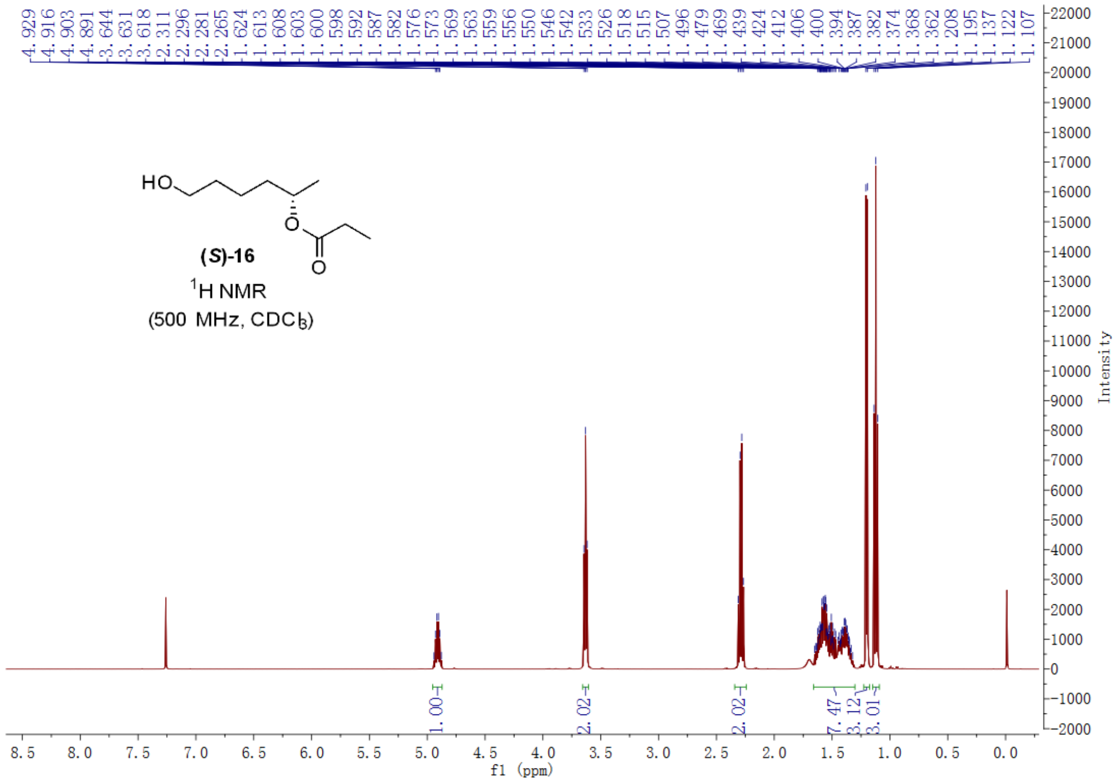
HO
O
O
(S)-16
¹H NMR
(500 MHz, CDCl3)
4.929
4.916
4.903
4.891
3.644
3.631
3.618
2.311
2.296
2.281
2.265
1.00
2.02
2.02
7.47
3.12
3.01
f1 (ppm)
Intensity

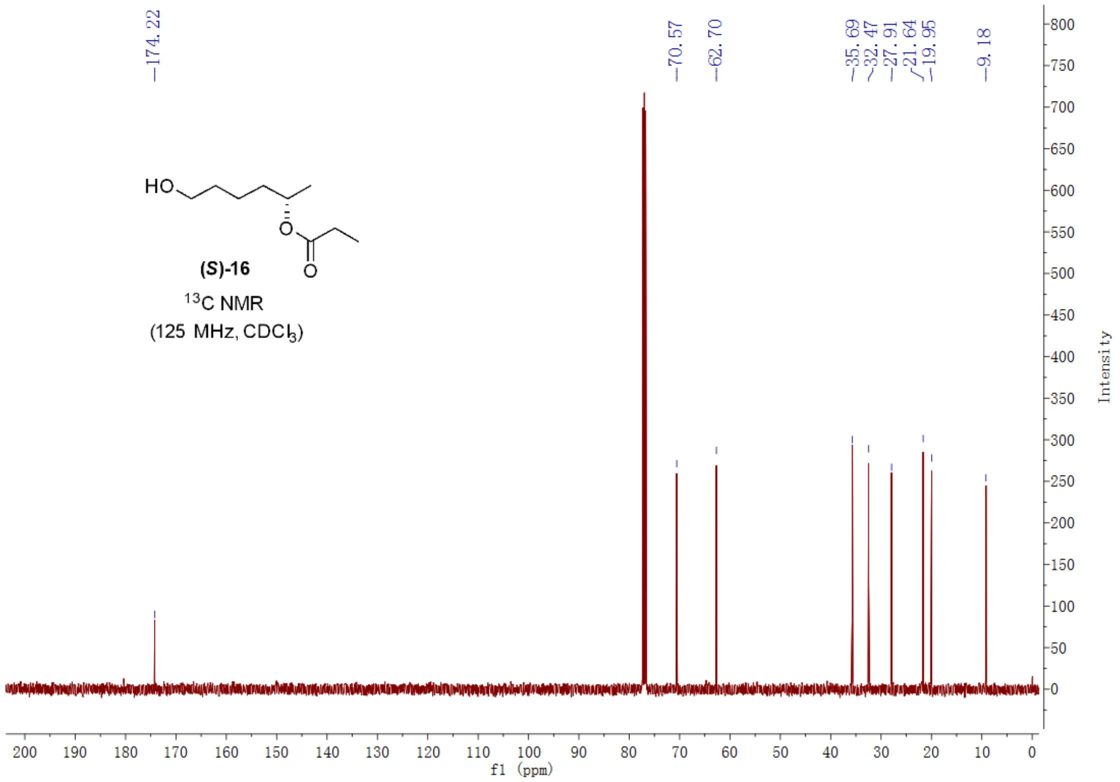
HO
O
O
(S)-16
¹³C NMR
(125 MHz, CDCl3)
174.22
70.57
62.70
35.69
32.47
27.91
21.64
19.95
9.18
f1 (ppm)
Intensity

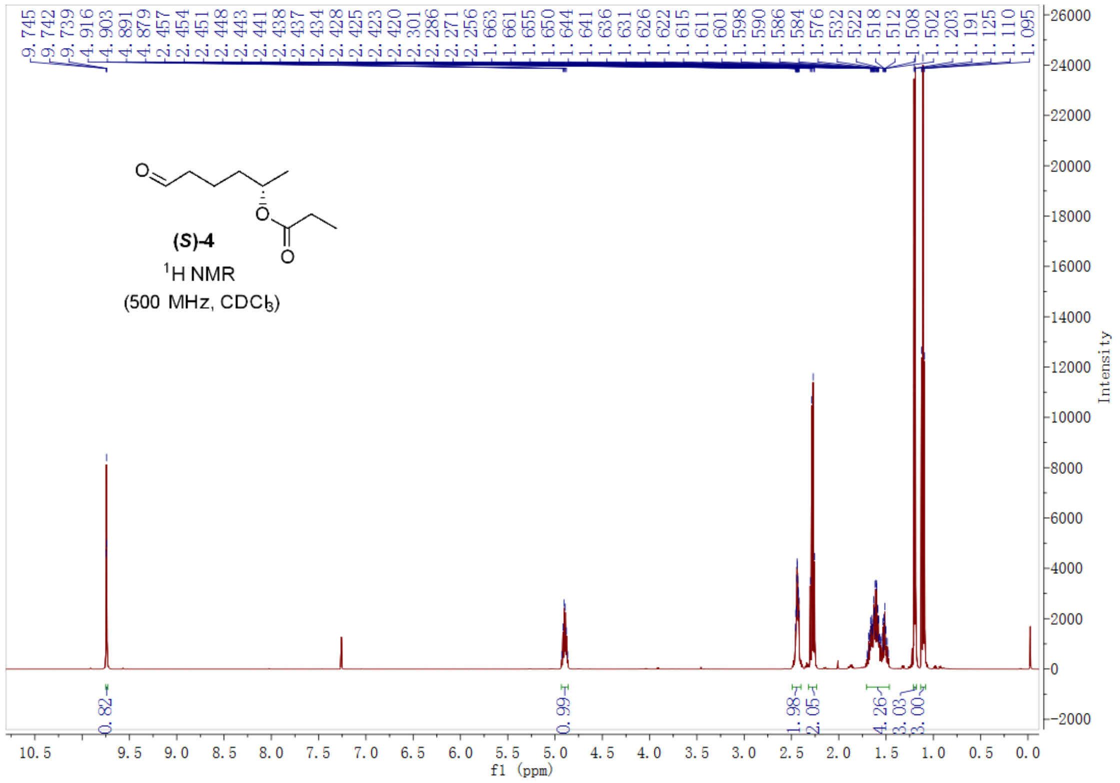

(S)-4
^{1}H NMR
(500 MHz, CDCl$_3$)
f1 (ppm)
Intensity

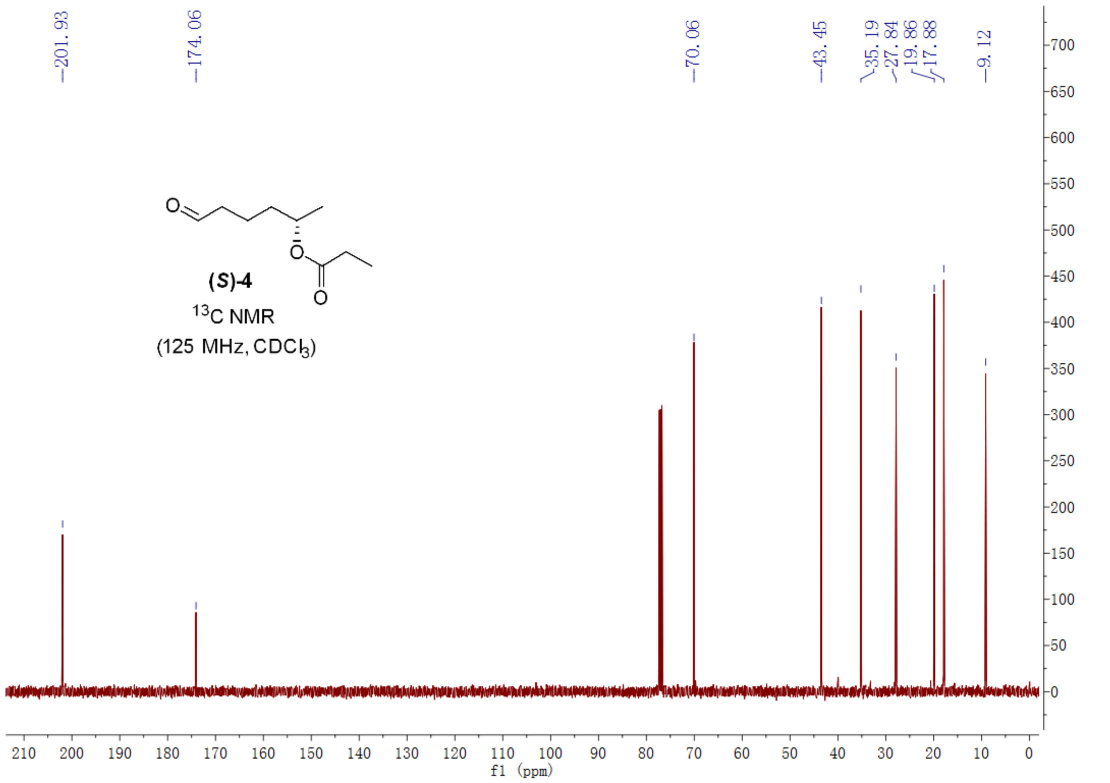

(S)-4
^{13}C NMR
(125 MHz, CDCl$_3$)
f1 (ppm)

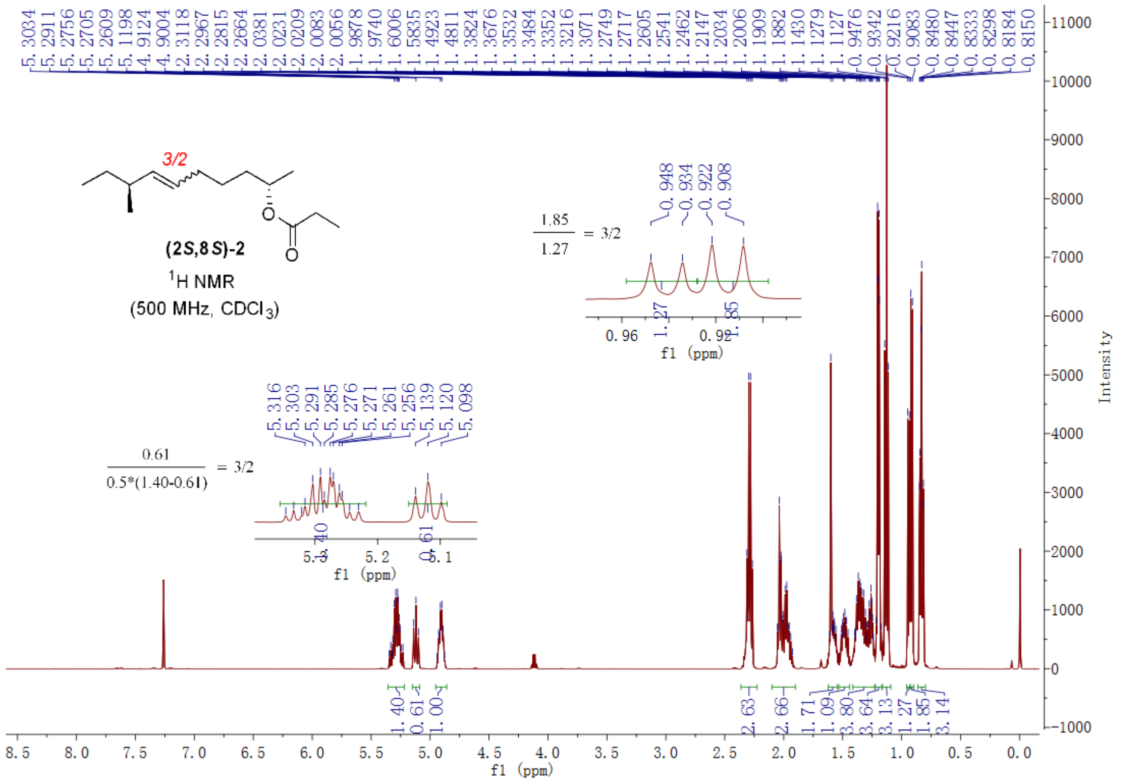
(2S,8S)-2
^{1}H NMR
(500 MHz, $CDCl_3$)
3/2
1.85 / 1.27 = 3/2
0.61 / 0.5*(1.40-0.61) = 3/2
f1 (ppm)
Intensity

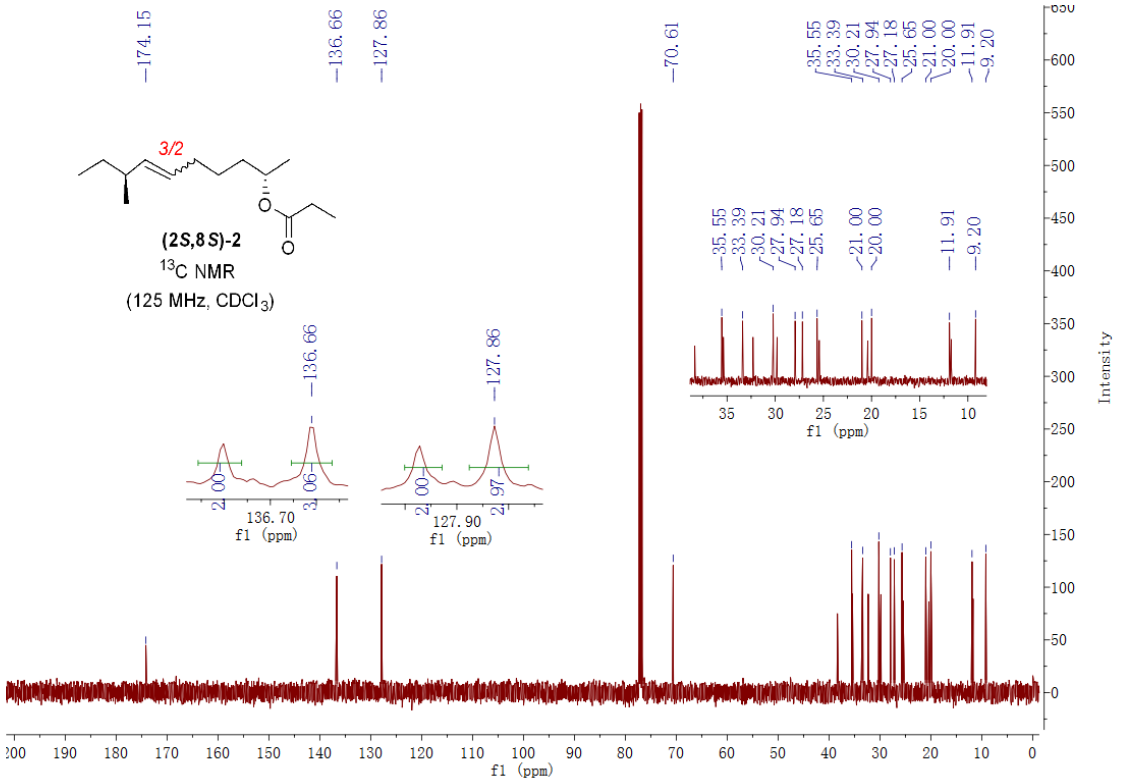
(2S,8S)-2
^{13}C NMR
(125 MHz, $CDCl_3$)
3/2
174.15
136.66
127.86
70.61
f1 (ppm)
Intensity

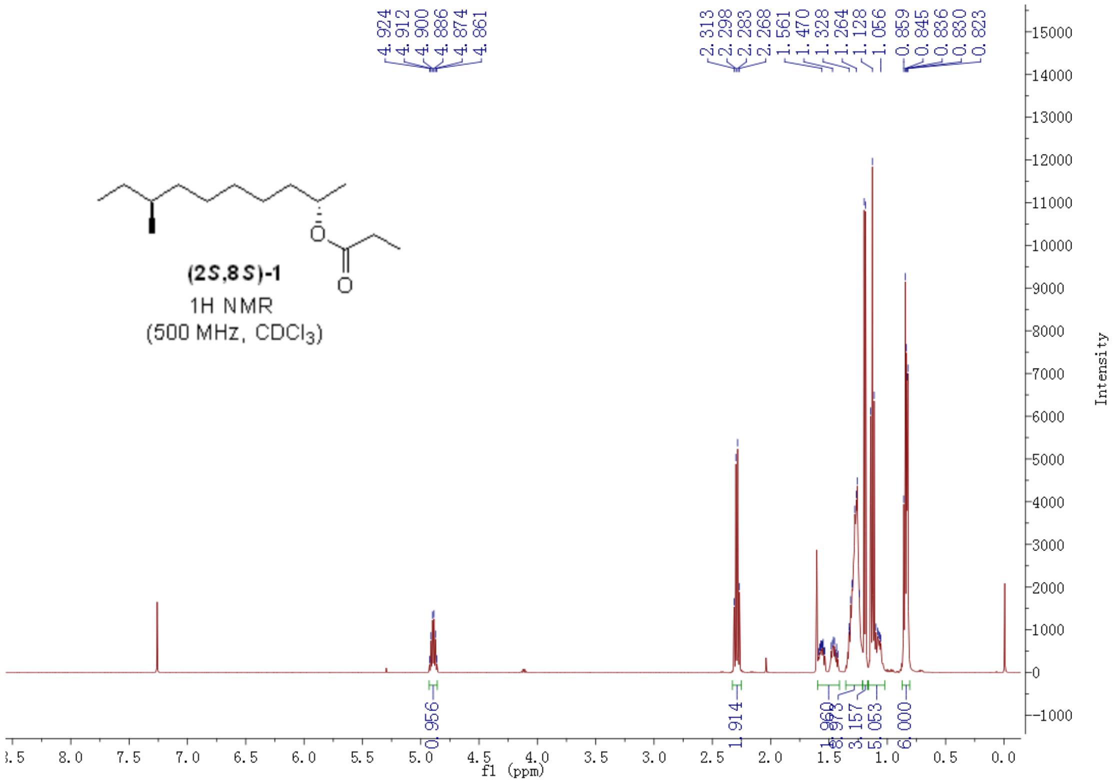

(2S,8S)-1
1H NMR
(500 MHz, CDCl3)
4.924
4.912
4.900
4.886
4.874
4.861
2.313
2.298
2.283
2.268
1.561
1.470
1.328
1.264
1.128
1.066
0.859
0.845
0.836
0.830
0.823
f1 (ppm)
Intensity

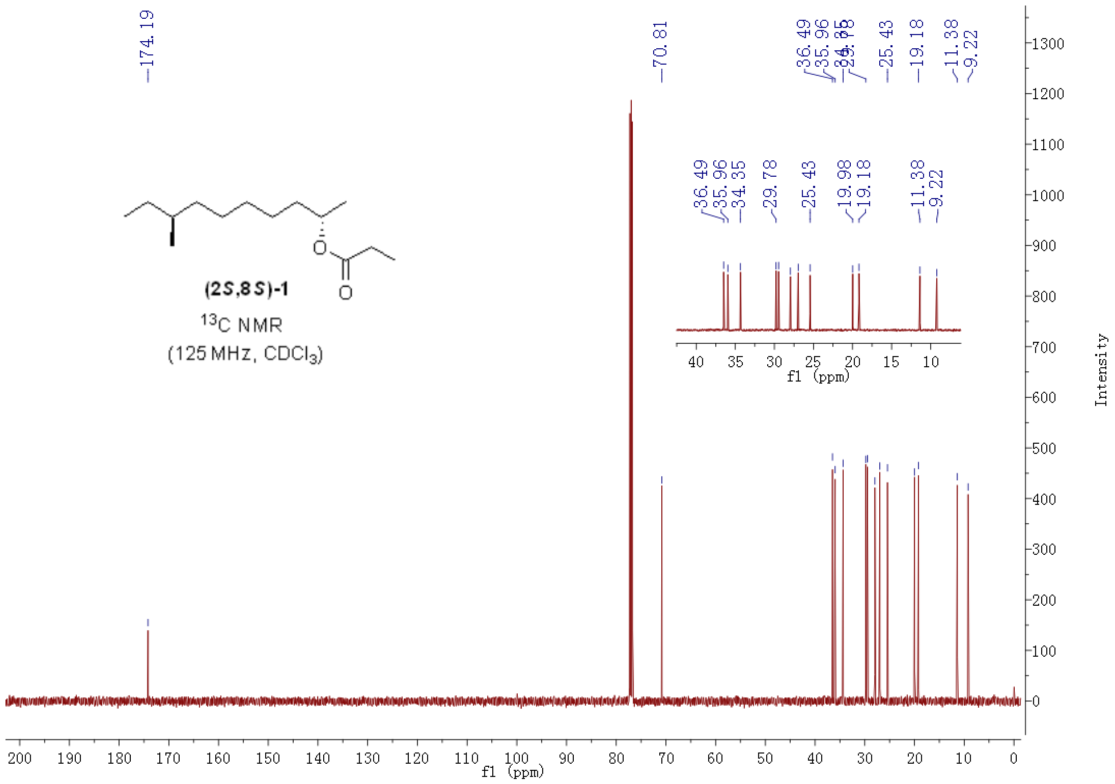

(2S,8S)-1
13C NMR
(125 MHz, CDCl3)
174.19
70.81
36.49
35.96
34.35
29.78
25.43
19.98
19.18
11.38
9.22
f1 (ppm)
Intensity

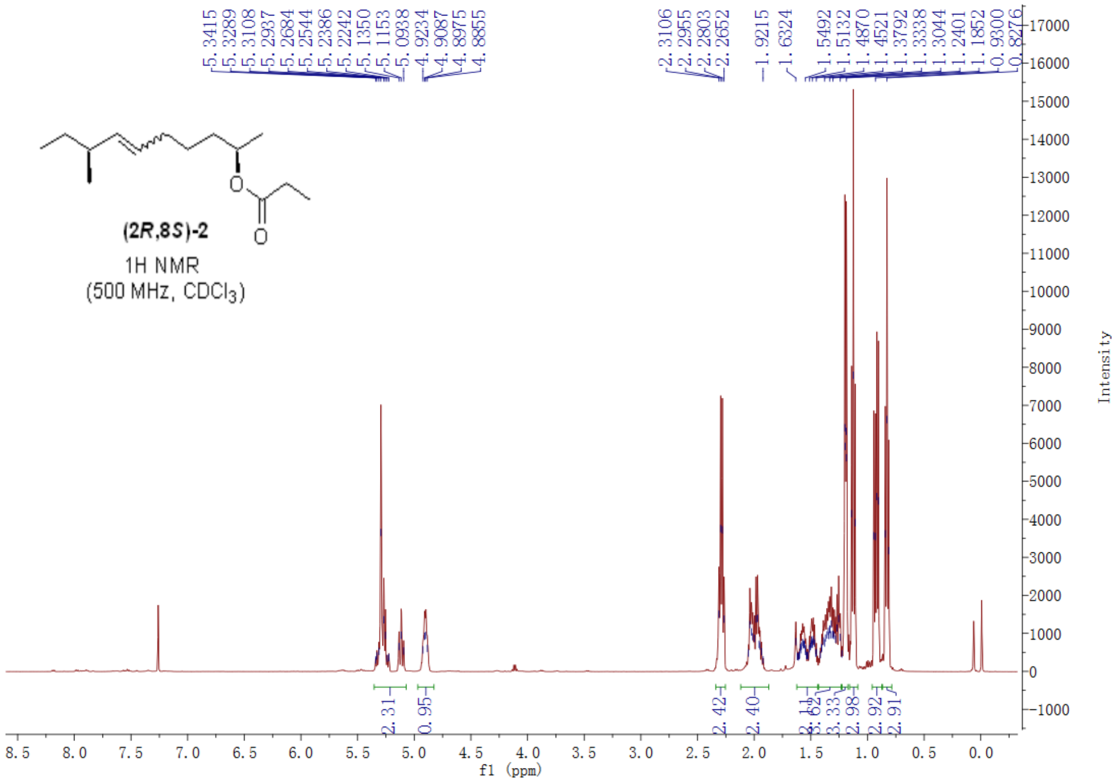

(2R,8S)-2
1H NMR
(500 MHz, $CDCl_3$)
f1 (ppm)
Intensity

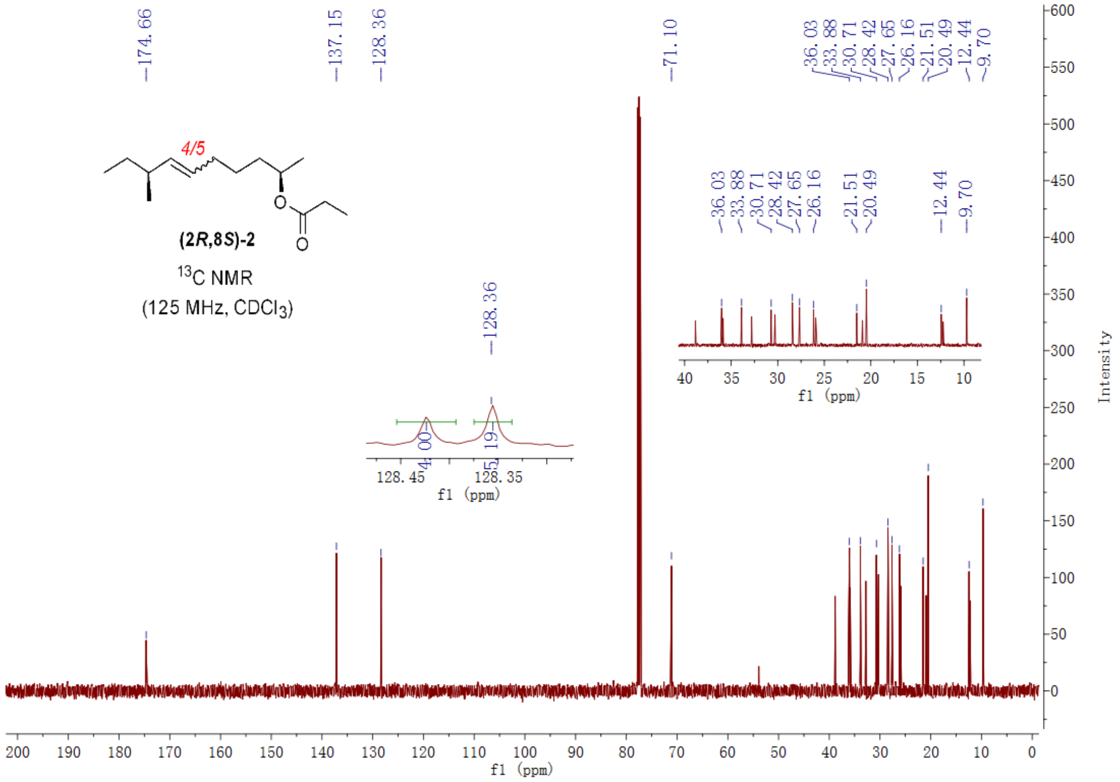

4/5
(2R,8S)-2
^{13}C NMR
(125 MHz, $CDCl_3$)
174.66
137.15
128.36
71.10
36.03
33.88
30.71
28.42
27.65
26.16
21.51
20.49
12.44
9.70
f1 (ppm)
Intensity

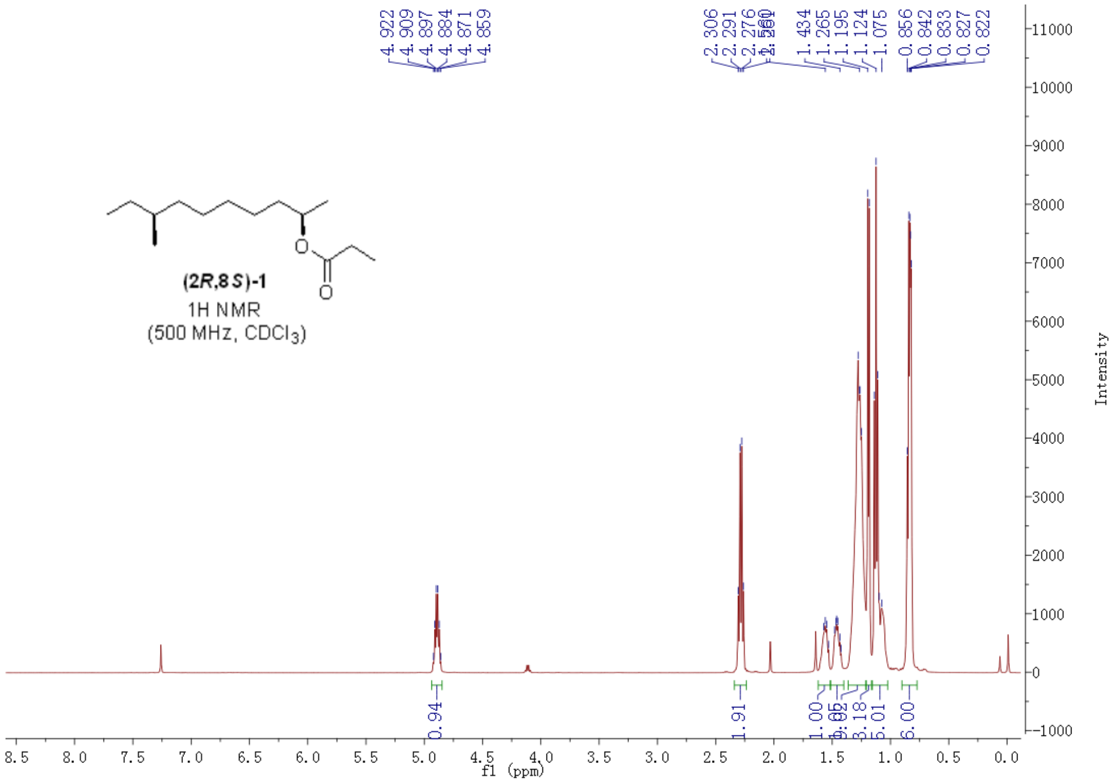
(2R,8S)-1
1H NMR
(500 MHz, CDCl3)
4.922 4.909 4.897 4.884 4.871 4.859
2.306 2.291 2.276
1.434 1.265 1.195 1.124 1.075
0.856 0.842 0.833 0.827 0.822
0.94
1.91
1.00
3.18
5.01
6.00
f1 (ppm)
Intensity

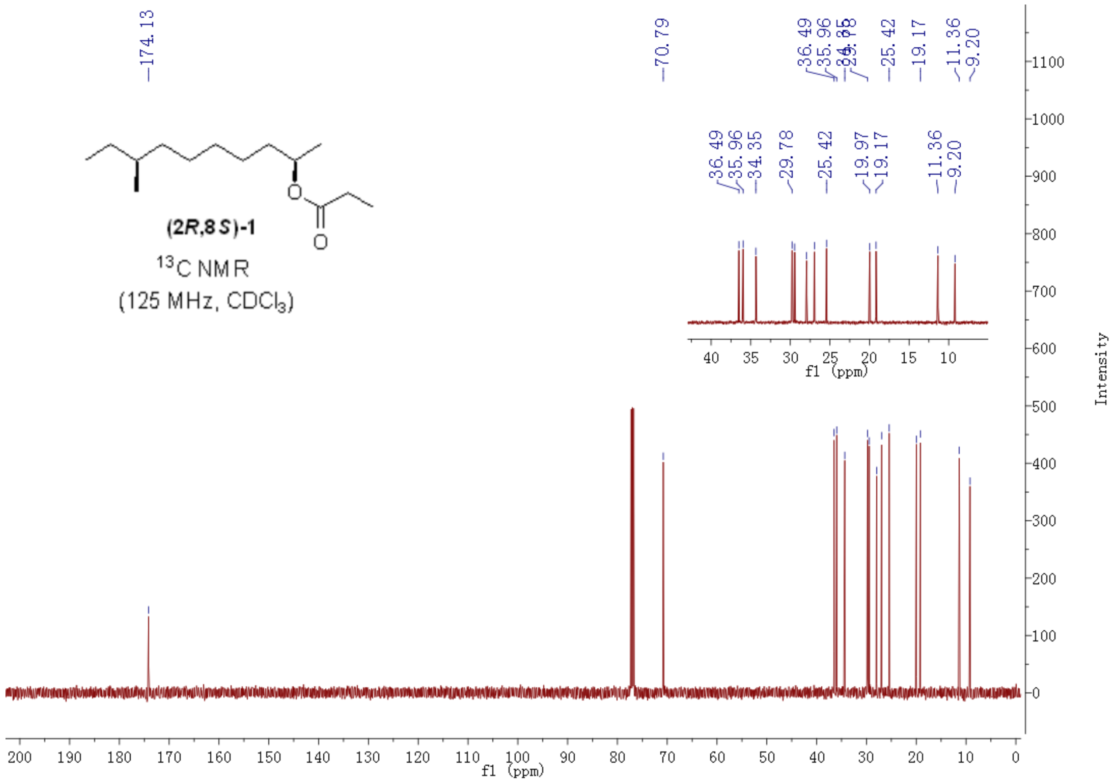
(2R,8S)-1
13C NMR
(125 MHz, CDCl3)
174.13
70.79
36.49 35.96 34.35 29.78 25.42 19.97 19.17 11.36 9.20
f1 (ppm)
Intensity

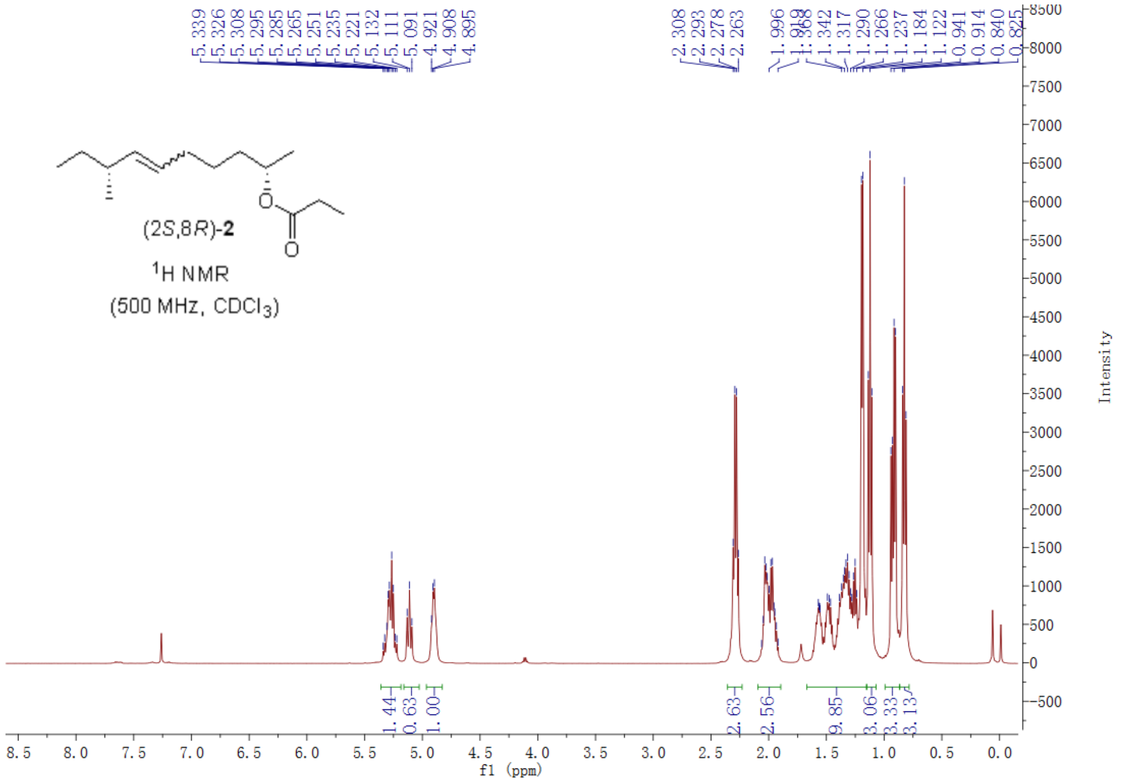

(2S,8R)-2
1H NMR
(500 MHz, CDCl3)
5.339
5.326
5.308
5.295
5.285
5.265
5.251
5.235
5.221
5.132
5.111
5.091
4.921
4.908
4.895
2.308
2.293
2.278
2.263
1.996
1.342
1.317
1.290
1.266
1.237
1.184
1.122
0.941
0.914
0.840
1.44
0.63
1.00
2.63
2.56
9.85
3.06
3.33
3.13
f1 (ppm)
Intensity

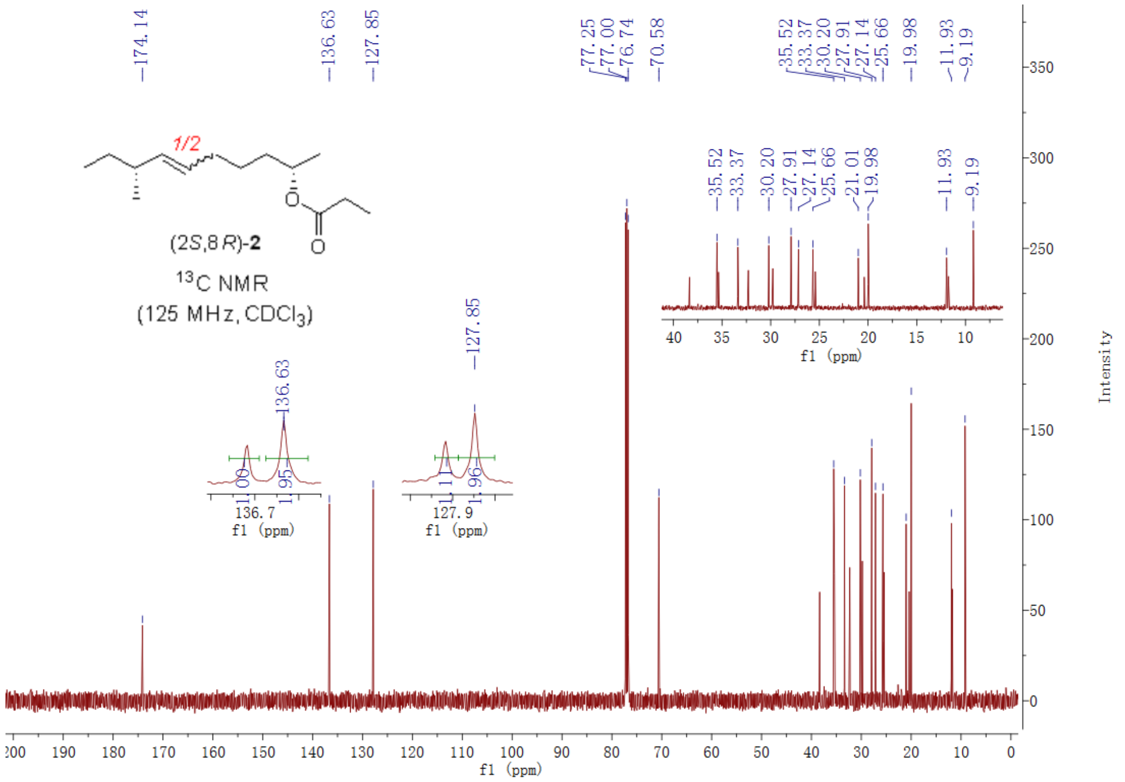

1/2
(2S,8R)-2
13C NMR
(125 MHz, CDCl3)
174.14
136.63
127.85
77.25
77.00
76.74
70.58
35.52
33.37
30.20
27.91
27.14
25.66
21.01
19.98
11.93
9.19
1.00
1.95
136.7
1.11
1.96
127.9
f1 (ppm)
Intensity

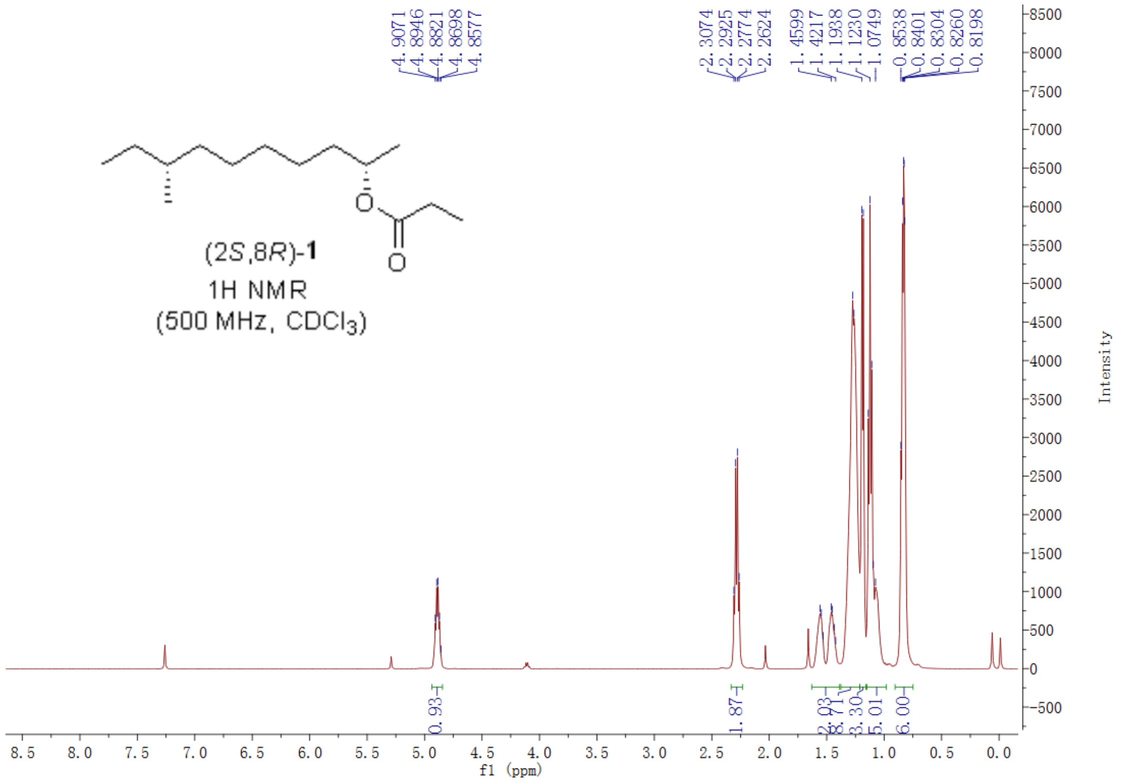
(2S,8R)-1
1H NMR
(500 MHz, CDCl3)
Intensity
f1 (ppm)

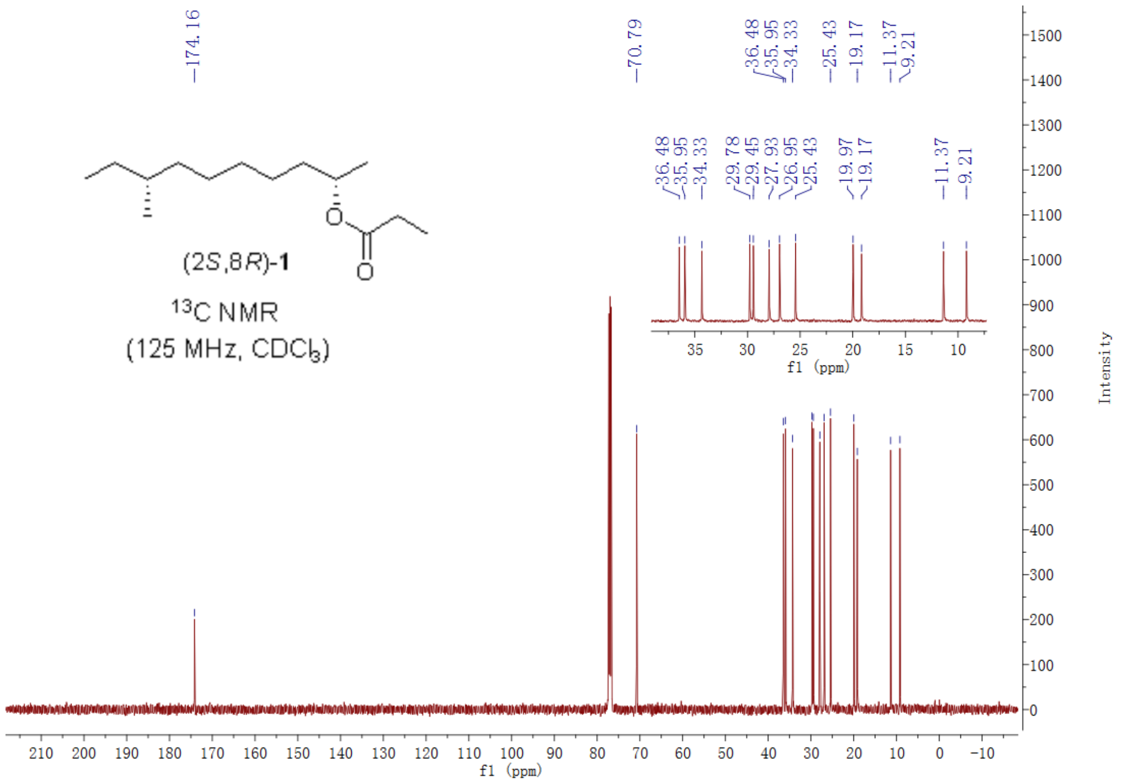
(2S,8R)-1
13C NMR
(125 MHz, CDCl3)
Intensity
f1 (ppm)

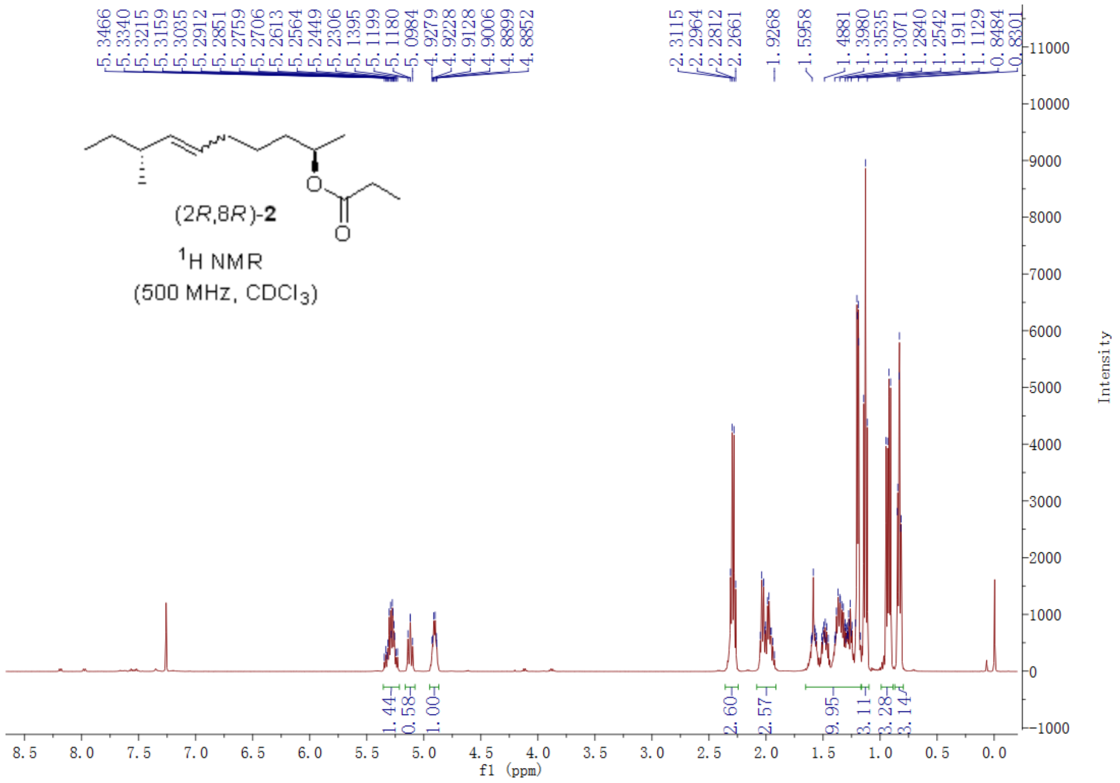
(2R,8R)-2
1H NMR
(500 MHz, CDCl3)

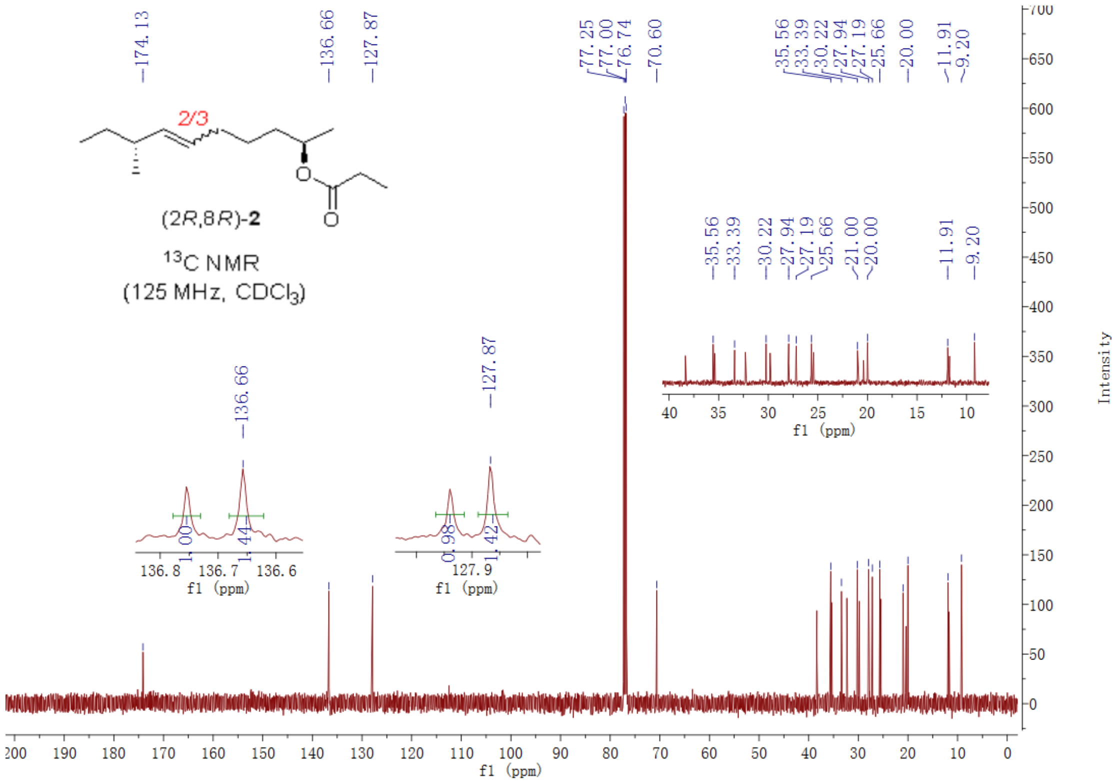
2/3
(2R,8R)-2
13C NMR
(125 MHz, CDCl3)

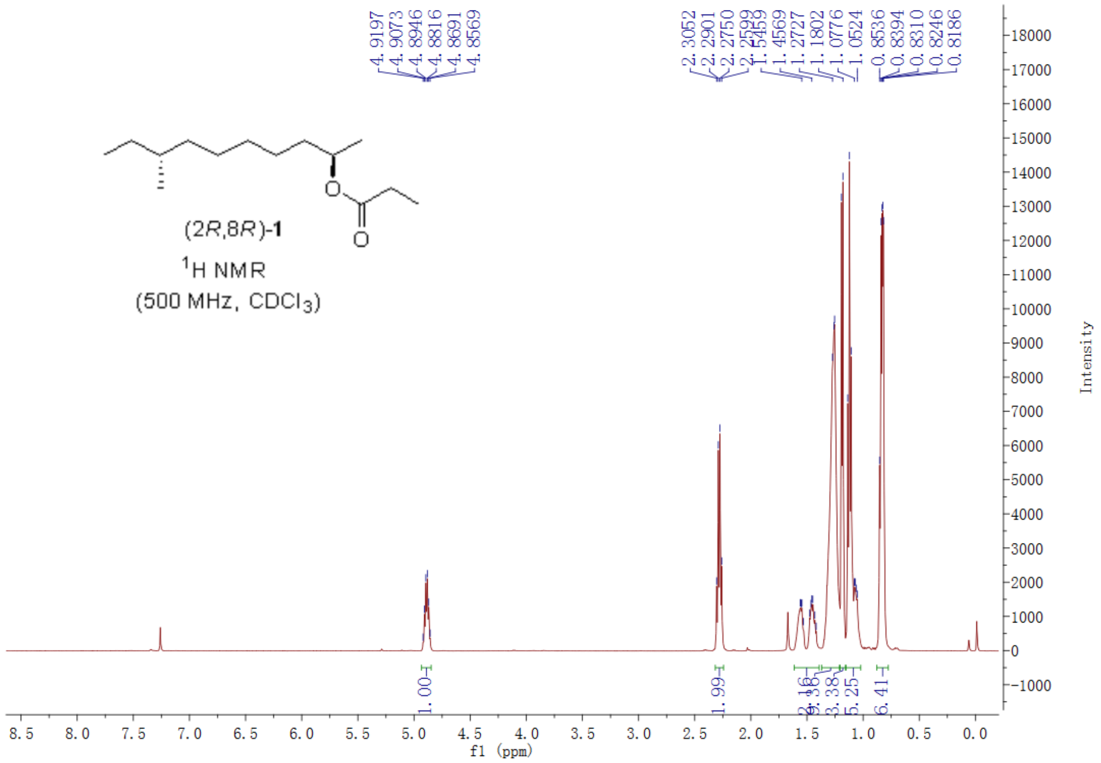
4.9197
4.9073
4.8946
4.8816
4.8691
4.8569
2.3052
2.2901
2.2750
1.4569
1.2727
1.1802
1.0776
1.0524
0.8536
0.8394
0.8310
0.8246
0.8186
(2R,8R)-1
1H NMR
(500 MHz, CDCl3)
1.00
1.99
5.25
6.41
f1 (ppm)
Intensity

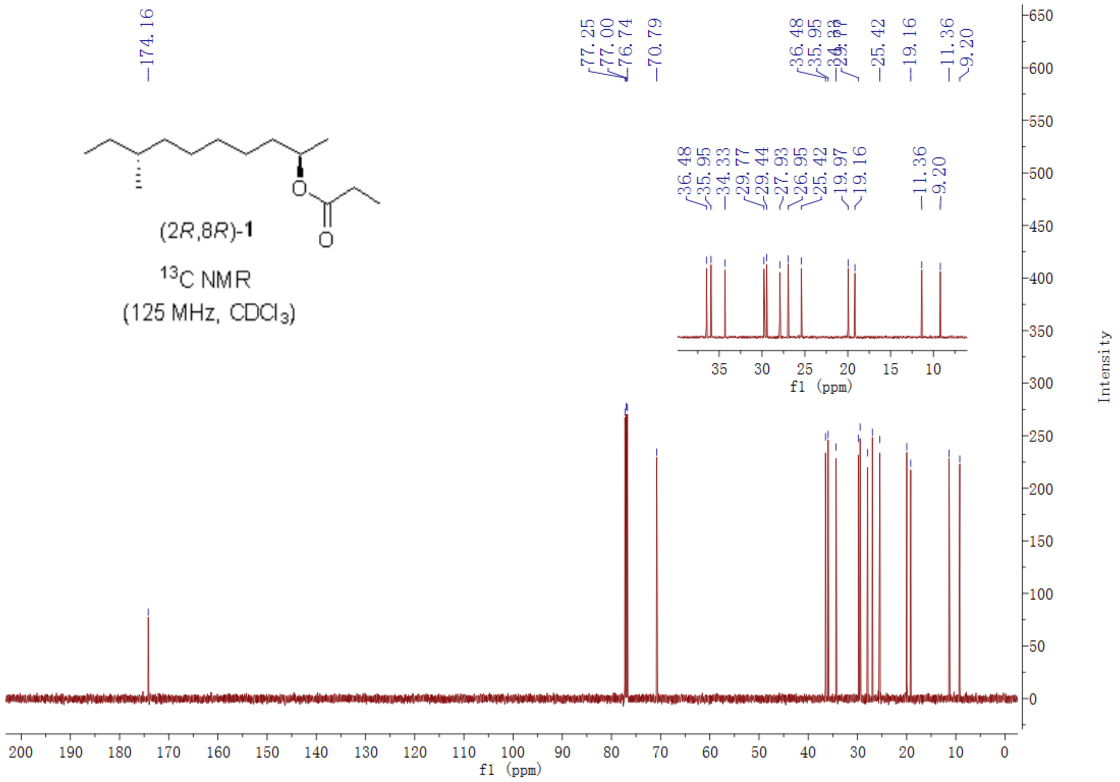
174.16
77.25
77.00
76.74
70.79
36.48
35.95
25.42
19.16
11.36
9.20
(2R,8R)-1
13C NMR
(125 MHz, CDCl3)
36.48
35.95
34.33
29.77
29.44
27.93
26.95
25.42
19.97
19.16
11.36
9.20
f1 (ppm)
Intensity
